# Detection of Organelle-Specific Dyes Labeled Extracellular Vesicles with Colocalization-Fluorescence Nanoparticle Tracking Analysis

**DOI:** 10.1021/acsomega.5c12069

**Published:** 2026-02-03

**Authors:** Getnet Midekessa, Kasun Godakumara, Mohammad Mehedi Hasan, Aneta Andronowska, Alireza Fazeli

**Affiliations:** † Department of Pathophysiology, Institute of Biomedicine and Translational Medicine, University of Tartu, Tartu 50411, Estonia; ‡ Institute of Veterinary Medicine and Animal Sciences, 85334 Estonian University of Life Sciences, Tartu 51006, Estonia; § Research Department of Maternal and Fetal Medicine, Elizabeth Garrett Anderson Institute for Women's Health, University College London, London WC1E 6HX, U.K.; ∥ Institute of Animal Reproduction and Food Research, Polish Academy of Sciences, Olsztyn 10-683, Poland; ⊥ Academic Unit of Reproductive and Developmental Medicine, Department of Oncology and Metabolism, Medical School, University of Sheffield, Sheffield S10 2SF, U.K.

## Abstract

Most cell types release a diverse array of extracellular vesicles (EVs) that contribute to intercellular communication. In particular, considering the heterogeneity of EVs, methods capable of identifying and measuring individual vesicles are limited. Here, we used fluorescence and colocalization Nanoparticle Tracking Analysis (NTA) to identify the subcellular origin of vesicles and determine their physical characteristics, as well as colocalization ratios of endoplasmic (ER) and Mitochondria (Mito) positive EVs in human choriocarcinoma cells (JAr) and bovine follicular fluids (BFF). The labeling efficiency for ER-labeled JAr EVs purified in SEC was 67.11 ± 25.40%, compared to 96.27 ± 13.72% of BFF EVs. Regarding Mito dye labeling efficiency, SEC-purified BFF EVs (14.21 ± 7.45%) provided lower Mito-positive fluorescent particles than JAr EVs (25.74 ± 4.46%). The proportion of CellMask Deep-Red (CMDR) membrane labeling of nanoparticles varied across JAr and BFF EVs. Furthermore, colocalization analysis of ER and Mito-dye-labeled JAr and BFF revealed potential intracellular interactions between organelles and the EV biogenesis pathways. The integration of novel colocalization technology into fluorescence-NTA (F-NTA) represents a significant advancement in the field of single EV particle analysis in deepening our understanding of EV biology.

## Graphical abstract

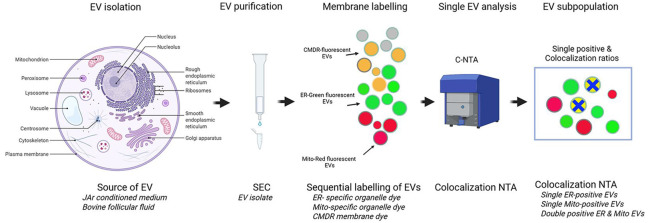

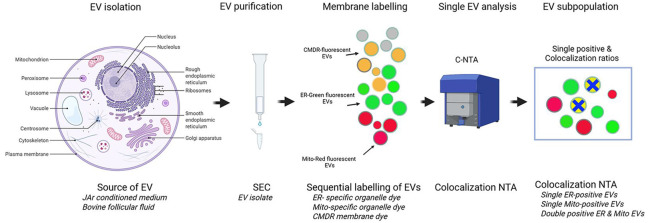

## 1 Introduction

EVs are membrane-bound particles containing biologically active and highly heterogeneous cargo.^1^ Most cell types release EVs into the extracellular space and can be present in various biological fluids and cell culture conditioned medium.^2,3^ The past decades have witnessed the rapid development of EV roles in mediating intercellular communications, being vectors for drug delivery vehicles and potential disease biomarkers.^4–7^ Owing to these unique attributes, the detection and characterization of EVs derive biological insights and contribute to their multifaceted applications. EVs are commonly classified into different subtypes (exosomes and microvesicles) based on their multiple biogenesis and site of subcellular origins, which vary greatly in size, surface protein profiles, and cargo composition.^8,9^ The heterogeneity nature of EVs is crucial in understanding their unique biological roles in different physiological and pathological processes.^10–12^ However, it is extremely challenging to analyze such heterogeneous EVs on a single-particle basis, suggesting a need for multiple platforms to characterize them.

The biogenesis of EVs is still largely unknown, but it has been established that they are generated via at least two distinct mechanisms. EVs are formed through the endosomal pathway via invagination of the plasma membrane to be secreted called exosomes.^13^ The biogenesis and secretion of exosomes involve a coordinated process that is driven by an endosomal sorting complex required for transport (ESCRT) dependent and ESCRT-independent pathways.^14,15^ On the other hand, EVs can originate from outward budding followed by blebbing of the plasma membrane to produce microvesicles.^16^ These vesicles vary in size and molecular composition. For instance, exosomes are the smallest EVs with diameters of ≤200 nm, whereas microvesicles have a broad size range (100–1000 nm) during biogenesis.^15^

Beyond known EV biogenesis sites, EV characterization questions related to the EV heterogeneity originating from different cellular organelle-derived vesicles remain unanswered. The intracellular trafficking of EVs after endosomal escape is a complex biological process involving multiple cellular structures, organelles, and molecules. For instance, Barman et al. revealed that endoplasmic reticulum (ER) membrane contact site linker protein, named vesicle-associated protein A (VAP-A), drives biogenesis of a subset of RNA-enriched EVs.^17^ Recent studies have also shown that lysosomal inhibition leads to increased secretion of mitochondria in large EVs, indicating a pathway for the removal of dysfunctional mitochondria from cells.^18,19^ These and other evidence highlight the need to consider the entire trafficking landscape of intracellular vesicular organelles that can directly, or indirectly, contribute to EV biogenesis and secretion.^20,21^ Such variations related to EV heterogeneity, however, have not been evaluated in single EV level previously.

With major advances in single EV analysis methods, it is now possible to study EV heterogeneity in a given sample. This is shown in the increasing use of fluorescent membrane probes/dyes for EV labeling and monitoring the double-layered lipid membrane of EVs using different single EV detection platforms.^22–25^ NTA, being the most known quantitative method for EV analysis, uses captured scattering light of individual particles to determine the size or/and concentrations of EVs.^26^ With the introduction of fluorescence into NTA, it can be used to distinguish particles of EV’s origin and those particles that are not of EV origin by targeting their prominent characteristics.

Given the distinct mechanisms for EV biogenesis and secretion,^27^ we hypothesized that vesicles can also originate from different cellular organelles such as the ER, mitochondria (Mito), and others. Thus, they may also contribute to heterogeneous populations of EVs. In the present study, we used three fluorescent membrane probes specific to ER, Mito, and CMDR to characterize and identify EV populations from different origins. ER and Mito dyes were used to label organelle-derived vesicles. CellMask Red dye stained the EV membrane and confirmed the purity of the size-exclusion chromatography (SEC)-purified JAr and bovine follicular fluid (BFF) EVs. Conditioned media and biological fluids have been widely utilized for EV isolation in biological studies. We employed two well-established experimental models routinely used in our laboratory to ensure reproducibility and methodological consistency. The human choriocarcinoma JAr cell line served as an in vitro model for investigating trophoblast differentiation and embryo–maternal communication.^28^ In addition, follicular fluid, an ovarian fluid supporting spermatozoa maturation, was used as a biologically relevant in vivo-derived sample.^29^ The selection of these systems minimized the potential variability associated with unfamiliar experimental models. Furthermore, we assessed the effect of those dyes on the physical characteristics of labeled vesicles using fluorescence-NTA. The difference in the physical properties between ER- and Mito-labeled nanoparticles (NPs) in fluorescence and scattering modes was evaluated. Furthermore, we identified the multiple fluorescence signals of individual EVs and analyzed the colocalization of ER- and Mito-positive EVs using colocalization-NTA (C-NTA). Throughout this paper, the terms “total nanoparticles” (t-NPs) and ‘fluorescent nanoparticles’ (fl-NPs) refer to particles detected in the scattering and fluorescence modes of NTA, respectively.

## 2 Materials and Methods

### 2.1 Materials

Dulbecco’s PBS (Sigma-Aldrich Co., St. Louis, MO), CellMask Deep-Red plasma membrane stain (Catalog number: C10046, Thermo Fisher Scientific, Eugene, OR), PhenoVue Fluor 488—Concanavalin A (Catalog number: CP94881, PerkinElmer, Shelton, CT), and MitoLite Deep-Red FX660 (Catalog number: 22678, AAT Bioquest, Pleasanton, CA).

### 2.2 Cell Culture

The human choriocarcinoma cell line (JAr) from the first-trimester trophoblasts was acquired from ATCC 116 (HTB-144, Teddington, UK). The human choriocarcinoma (JAr) cells were cultured as described previously.^28^ Briefly, JAr cells were cultured in a T-75 flask in RPMI 1640 media (Gibco, Paisley, Scotland) supplemented with 1% penicillin/streptomycin (P/S, Gibco 15140122, Bleiswijk, The Netherlands), 1% l-glutamine (Sigma, 59202C, St. Louis, MO), and 10% fetal bovine serum (FBS, Gibco, 10500064) at 37 °C under moist 5% CO_2_-rich conditions. At 80% confluency, the conditioned medium was removed, and the cells were washed with 10 mL of unsupplemented RPMI 1640 media to remove traces of FBS. Unsupplemented RPMI medium was replaced with fresh RPMI 1640 medium supplemented with 1% penicillin/streptomycin, 1% l-glutamine, and 10% EV-depleted FBS. Cells were cultured for 24 h at 37 °C and 5% CO_2_. After incubation, the conditioned medium was collected for EV isolation. We have submitted all relevant data of our experiments to the EV-TRACK knowledgebase (EV-TRACK ID: EV190091).^30^

#### 2.2.1 EV-Depletion of the FBS

The depletion of EVs in FBS was carried out using a methodology proposed earlier.^31^ In brief, FBS was ultrafiltered using Amicon Ultra-15 centrifugal filter devices (100 kDa cutoff, Merk Millipore, Darmstadt, Germany) for 30 min at 5000*g*. The filtrate was collected and measured for particle concentration using NTA. For this study, the vesicle-depleted conditioned medium was used to isolate and purify EVs by SEC, and the EV preparations met the optimal requirement as per the guidelines prescribed by the International Society for Extracellular Vesicles (ISEV).^9^

### 2.3 EV Purification from JAr Cell Conditioned Medium

Isolation of EVs by size-exclusion chromatography (SEC) was performed based on prior published methods.^32^ The conditioned medium was first spun at 400*g* for 10 min, and the supernatant from two successive centrifugation steps was retained (4000 and 10,000*g* for 10 min) to remove cell debris and apoptotic bodies. The collected conditioned medium was concentrated to 500 μL with Amicon Ultra-15 centrifugal filter devices (10 kDa cutoff, Merk Millipore, Darmstadt, Germany), and EVs were isolated from the concentrated media using SEC in a cross-linked 4% agarose matrix of 90 μm beads (Sepharose 4 Fast Flow, GE HealthCare Bio-Sciences AB, Uppsala, Sweden) in a 10 cm gravity column (Econo-Pac Chromatography columns, Bio-Rad, Hercules, CA), washed, and calibrated with PBS. The EV-enriched fractions 6 to 9 (volume 0.5 mL) were collected and concentrated further using Amicon Ultra-15 centrifugal filter devices (10 kDa cutoff), as described previously.^28^ Isolated EVs were quantified using NTA (ZetaView, Particle Metrix GmbH, Inning am Ammersee, Germany). Proteomic and electron microscopic characterizations of JAr EVs were published in earlier communications.^28,32^

### 2.4 EV Purification from Bovine Follicular Fluid

The extracellular vesicles from bovine follicular fluid (BFF) were purified based on methods described in the literature.^33^ In brief, EVs were isolated from the follicular fluid of ovaries (BFF) obtained from a slaughterhouse (Rakvere, Estonia). Initially, the ovaries were washed three times using PBS supplemented with 1% penicillin–streptomycin and 1% amphotericin B. Then, the BFF was aspirated using a vacuum pump (Minitüb GmbH, Tiefenbach, Bavaria, Germany). Initially, samples were centrifuged at 300*g* for 10 min to remove cells. Subsequently, the supernatant from the previous step was centrifuged at 2000 and 20,000*g* for 10 and 30 min to remove the cell debris and apoptotic bodies from the follicular fluid. Later, the samples were concentrated up to 500 μL using Amicon Ultra-15 centrifugal filter units (10 kDa cutoff) (Merck Millipore, Burlington, MA) at 3000*g* for 1 h at 4 °C. Benchtop SEC columns were used to purify vesicles from the follicular fluid, as they were used for the isolation of JAr EVs. The vesicle-enriched fractions 5–7 (500 μL each) were collected and concentrated using Amicon Ultra-15 centrifugal filter units (10 kDa cutoff).

### 2.5 Organelle-Specific Labeling of EVs

#### 2.5.1 JAr and BFF EVs Labeling with ER Dye

JAr and BFF EVs purified in SEC were diluted separately in 1× PBS to a particle concentration of about 1 × 10^10^ particles/mL. Before incubating both EVs with ER dye molecules, 1 μL of 19.2 μM stock of PhenoVue Fluor 488— Concanavalin A (Catalog number: CP94881, PerkinElmer, Shelton, CT) was added to 40 μL of PBS. Then, 1 μL of ER prediluted in 1× PBS was added to 10 μL of diluted EVs and incubated at RT for 2 h. Additionally, by keeping the concentration of EVs constant, different concentrations of ER dye (384, 640, and 960 nM) were prepared following the same overall procedure in 1 × PBS to determine the optimum ER dye concentration. All experimental tubes were kept covered with aluminum foil during incubation. After incubation, the incubated samples were added to 990 μL of 1× PBS suspension medium to have a final volume of 1 mL with a pH value of 7.2.

#### 2.5.2 JAr and BFF EVs Labeling with Mitochondria Dye

JAr and BFF EVs purified in SEC were diluted separately in 1× PBS to a particle concentration of about 1 × 10^10^ particles/mL. Before incubating both EVs with Mito dye molecules, 1 μL of stock of MitoLite Deep-Red FX660 (Catalog number: 22678, AAT Bioquest, Pleasanton, CA) was added to 50 μL of PBS. Then, 1 μL of Mito in 1× PBS was added to 10 μL of diluted EVs and incubated at RT for 2 h. Here, we have prepared and tested different concentrations of Mito dye (30, 40, and 70×) following the same overall procedure in 1× PBS to determine the optimum Mito dye concentration. All experimental tubes were kept covered with aluminum foil during incubation. After incubation, the incubated samples were added to 990 μL of 1× PBS suspension medium to have a final volume of 1 mL with a pH value of 7.2.

#### 2.5.3 Sequential Labeling of JAr and BFF EVs with Mitochondria and ER Dyes

JAr and BFF EVs purified in SEC were diluted separately in 1× PBS to a particle concentration of about 1 × 10^10^ particles/mL. Ten microliters of diluted JAr EVs was sequentially colabeled with 50× MitoLite Deep-Red FX660 (Catalog number: 22678, AAT Bioquest, Pleasanton, CA) dye and 480 nM ER PhenoVue Fluor 488—Concanavalin A dye (Catalog number: CP94881, PerkinElmer, Shelton, CT) concentrations, whereas BFF EVs were sequentially colabeled with 40× Mito dye and 480 nM ER dyes and incubated at RT for 2 h. All experimental tubes were kept covered with aluminum foil during incubation. After incubation, the incubated samples were added to 990 μL of 1× PBS suspension medium to have a final volume of 1 mL with a pH value of 7.2.

#### 2.5.4 JAr and BFF EVs Labeling with CMDR Membrane Dye

JAr and BFF EVs purified in SEC were diluted separately in 1× PBS to a particle concentration of about 2 × 10^10^ particles/mL. Before incubating JAr and BFF EVs with CMDR dye molecules, 1 μL of 5 mg/mL CMDR stock CellMask Deep-Red plasma membrane stain (Catalog number: C10046, Thermo Fisher Scientific, Eugene, OR) was added to 2200 and 6000 μL of PBS, respectively. Then, 1 μL of CMDR in 1× PBS was added to 10 μL of diluted JAr and BFF EVs and incubated at RT for 2 h. Note that we tried different CMDR concentrations, such as 208.33, 238.1, and 243.9 ng/mL for JAr EVs and 50, 62.5, and 83.3 ng/mL for BFF EVs, to determine the optimum dye concentration. All experimental tubes were kept covered with aluminum foil during incubation. After incubation, the incubated samples were added to 1990 μL of 1× PBS suspension medium to have a final volume of 2 mL at a pH value of 7.2.

### 2.6 NP-40 Detergent Treatment of Neat (Untreated) and Fluorescent EVs

For detergent treatment of nanoparticles, EVs purified in SEC were diluted in 1× PBS to a particle concentration of about 2 × 10^10^ particles/mL. Before incubating EVs with and without ER, Mito, and CMDR dye molecules, the following dye concentrations were prepared for JAr and BFF EV samples in 1× PBS, respectively: 640/384 nM ER, 50*×*/40× Mito, and 227.27/83.3 ng/mL CMDR. Then, 1 μL of the respective dye in 1× PBS was sequentially added to 10 μL of diluted EVs for a total of 2 h RT incubation. Controls for detergent treatments included detergent only and EVs without detergent (NP-40). For NP-40 detergent treatment controls, EVs without or with respective dye(s) were treated with a final concentration of 0.5% NP-40 detergent in a 10 μL reaction volume. (Note: We tried different concentrations ranging from 0.01 to 2% of NP-40; among them, we found that EVs’ lipid membrane was disrupted and saturation was reached at 0.5% concentration of NP-40.) Samples were incubated for half an hour at RT (25 °C) following the addition of NP-40 detergent. Note that all experimental tubes were covered with aluminum foil during incubation. After incubation, the incubated samples were added to 1990 μL of 1× PBS suspension medium to have a final volume of 2 mL with a pH value of 7.2, resulting in a sample with final concentrations of about 1 × 10^8^ particles/mL EVs. The size and concentration of EVs were measured both in scatter and fluorescence modes as described below in the manuscript (Section 2.8).

### 2.7 Fluorescence Nanoparticle Tracking Analysis of EVs

Nanoparticle tracking analysis (NTA) was conducted using a ZetaView PMX-420 QUATT V4.3 instrument (Particle Metrix GmbH, Ammersee, Bavaria, Germany) equipped with four lasers, 405, 488, 520, and 640 nm, with the corresponding long-pass filters with 410, 500, 550, and 660 nm cutoff wavelengths. The instrument was auto-aligned using a known concentration of 100 nm polystyrene (PS), fluorescent PS Yellow-Green (YG) 488, and Deep-Red (DR) 660 beads (Applied Microspheres B.V., Leusden, Utrecht, The Netherlands). The standards were suspended in particle-free water, and the EV samples were diluted with 1× PBS for analyses. Particle number and size distribution were counted at 11 individual positions inside the measuring cell under a sensitivity of 72 and a shutter value of 100. The size and concentration measurements for fluorescently labeled EVs were measured at a sensitivity set at 90, while the data were analyzed by ZetaView NTA software version 8.05.14 SP7. Measurement parameters for NTA‘s scatter and fluorescence measurements are shown in Table S1. In an earlier communication, we also reported that fluorescent nanoparticles originating from JAr cells were dependent on the brightness threshold values set for the camera of the *fl*-NTA Instruments.^23^

### 2.8 Colocalization-Fluorescence Nanoparticle Tracking Analysis of ER- and Mito-Labeled EVs

ZetaView PMX-420 QUATT, equipped with ZetaNavigator software version 1.3.8.2, was used to obtain NTA measurements of size, concentration, and colocalization data. The instrument laser and microscope position for the selected filter and laser was auto-aligned using a known concentration of 100 nm TetraSpeck Microsphere polystyrene (PS), blue/green/orange/dark red (Catalog number: T7279, Thermo Fisher Scientific, Eugene, OR). In the ZetaNavigator software, the Shift-Pro tool was used to validate and correct all measurement positions with the TetraSpeck Microsphere Fluorescent PS with regard to identical illumination volumes of the fluorescence channels used. The standards were suspended in particle-free water, whereas the EV samples were diluted with 1× PBS for analyses. Colocalization experiments with a ZetaView NTA instrument involve an automated sequence of measurement steps in two fluorescence channels, for example, green and red. Short video sequences are recorded and imaged from the first and second channels, e.g., excitation with 640 and 488 nm lasers to excite red and green labeled particles, respectively. This step is followed by a colocalization event when a particle containing an overlay of both channels is detected within a certain Brownian range of motion. With the image processing, particles detected in red and green channels matched according to the nearest neighborhood criteria (i.e., link radius). The fast switching between the fluorescence channels and the software-defined link radius ensures a low distortion due to particle diffusion. In this study, one pair of fluorescence channels (488 and 640 nm) was used to determine the colocalization ratios of the EVs. Colocalization ratios are determined by dividing the number of colocalization events by the detections of channels ex640/F660 and ex488/F500, respectively. Measurement parameters for colocalization—NTA‘s scatter, fluorescence, and colocalization experiments—are shown in Table S2.

### 2.9 Transmission Electron Microscopy

JAr and BFF EVs for TEM imaging were prepared as described before with modifications.^28^ Briefly, 20 μL of the neat and NP-40-treated EV suspensions were deposited on Formvar-carbon-coated 200 mesh copper grids (Agar Scientific, Essex, U.K.) for 20 min. Next, EVs were contrasted for 2 min in 2% uranyl acetate (21447–25, Polysciences, Warrington) and air-dried for 10 min. The EVs were imaged using a JEM 1400 TEM instrument (JEOL Ltd., Tokyo, Japan, with Morada TEM CCD camera, Olympus, Germany) at 80 kV.

### 2.10 Statistical Analysis

Statistical analyses were performed using GraphPad Prism v8.4.2. Outliers were detected by Grubb’s test and excluded from the analysis using ZetaView NTA software. The comparison between the concentration and mean particle sizes of fluorescently labeled and total particles was assessed using the two-tailed Student *t*-test. The effects of different conditions were carried out using one- or two-way ANOVAs. Tukey’s multiple comparison tests were applied for specific intergroup comparisons. Data are shown as mean ± SD (*n* = 3). Differences were taken as statistically significant at *p* ≤ 0.05 and were marked with an asterisk (*) symbol.

### 2.11 Experimental Design

The experiments were designed to test the validity of the following hypotheses.

#### 2.11.1 ER and Mito Dye Concentrations Affect the Particle Size Distribution, Mean Size, and Concentration of ER- and Mito-Labeled Fluorescent EVs

Experiments were performed to determine the effect of ER and Mito organelle-specific dye concentration on the particle size and concentration of fluorescent nanoparticles (fl-NPs) compared to t-NPs present in a given sample. The effect of ER and Mito dye concentration on the physical characteristics of fl-NPs derived from the BFF and JAr cells was studied at a varied concentration while keeping the concentration of EVs constant. Experiments were performed in triplicate with 3 technical replicates. The size, concentration, and fluorescence of EVs were measured and determined at 25 °C (*n* = 9).

#### 2.11.2 Sequence of Labeling Affects the Detection of Fluorescent Nanoparticles

Experiments were performed to investigate whether the detection of fluorescent nanoparticles is affected by sequences of EV labeling. JAr and BFF EVs were colabeled and/or sequentially labeled with respective ER and Mito organelle-specific dyes. The effect of sequences of EV labeling on the physical properties of fl-NPs compared with t-NPs present in a given sample. Experiments were performed in triplicate, and the particle size distribution, concentration, and fluorescence of EVs and t-NPs were measured in scatter and fluorescence modes, as described elsewhere.

#### 2.11.3 Source of EVs Alters the Colocalization Ratio of ER and Mito Double-Positive Vesicles

Experiments were performed to identify the subcellular origin of vesicles derived from the ER and mitochondrion compartments of the cells. JAr and BFF EVs were sequentially colabeled with Mito and ER organelle-specific dyes and detected using Colocalization-NTA (C-NTA). C-NTA was used to determine the colocalization of vesicles derived from the ER and mitochondrion compartments of the cells, as well as single positive populations in the given sample. Experiments were performed in triplicate with 3 technical replicates. The size, concentration, fluorescence, and colocalization ratios of EVs were measured both in scatter and fluorescence modes of ZetaView C-NTA, as described elsewhere.

#### 2.11.4 Detergent Treatments of EV Membrane Affect the Proportion of Fluorescent NPs

Experiments were performed to confirm the disruption of the EV lipid bilayer membrane using a nonionic NP-40 detergent in the absence and presence of ER, Mito, and CMDR dyes within a given sample. Detergent treatment was performed for JAr and BFF EVs in both the absence and presence of the ER, Mito, and CMDR dyes. Experiments were performed in triplicate. The size and concentration of EVs during pre- and post-NP-40 detergent treatment, as well as NP-40-treated fl-NPs and t-NPs, were measured in scatter and fluorescence modes, as mentioned elsewhere.

The figure below illustrates the overall experimental workflow, including EV isolation using SEC, organelle dye labeling of EVs, and their detection using fluorescence and colocalization NTA, as well as TEM.

## 3 Results

### 3.1 Optimizing the Dye Concentration of ER- and Mito-Labeled JAr and BFF EVs

To evaluate the effect of ER and Mito dye concentration on the physical characteristics of EVs, JAr and BFF EVs were labeled without and with ER-specific organelle dyes at 384, 480, 640, and 960 nM, respectively. Both EV types were also labeled with Mito-specific organelle dye at 70, 50, 40, and 30× (Figure 1). By keeping EV concentration constant, the concentration of ER and Mito dyes varied accordingly. Heterogeneity in EV sizes was observed for JAr and BFF EVs labeled with ER (Figure S1A,B) and Mito (Figure S2A,B)-specific organelle dyes. ER and Mito-specific organelle dye concentration affected the size distribution of fluorescently labeled nanoparticles originating from BFF and JAr cells. The mean size of total and fluorescent nanoparticles derived from JAr cells (Figure S1C­(i)) was maintained at the two lowest ER dye concentrations, while fluorescent NPs of BFF (Figure S1D­(i)) showed a significant shift in the mean toward larger particle size (*p* < 0.05). Regarding Mito-labeled EVs, the mean size of total and fluorescent nanoparticles derived from JAr cells (Figure S2C­(i)) did not show a significant shift toward smaller particle size with successive increases in Mito dye concentration, while fluorescent NPs of BFF (Figure S2D­(i)) showed a significant shift in the mean toward larger particle size (*p* < 0.05) at 50× Mito dye concentration. Fluorescent NPs of BFF EVs at the lowest concentration of Mito dye were not detected using Quatt ZetaView NTA. Otherwise, successive increases in ER-specific organelle dye concentration led to smaller particle mean size and higher labeling of fluorescently labeled nanoparticles of JAr (Figure S1C­(ii)) and BFF EVs (Figure S1D­(ii)). A similar trend in higher labeling of fluorescently labeled nanoparticles of JAr (Figure S2C­(ii)) and BFF EVs (Figure S2D­(ii)) was also observed with successive increases in Mito-dye concentration. In all cases, ER and Mito nanoparticles were not detected in the respective negative ER and Mito only controls, in both the scatter and fluorescence modes of NTA. Overall, these results suggest prioritizing the optimization of dye concentrations to maintain the size profile between fluorescence and scatter modes of NTA.

**Figure 1:**
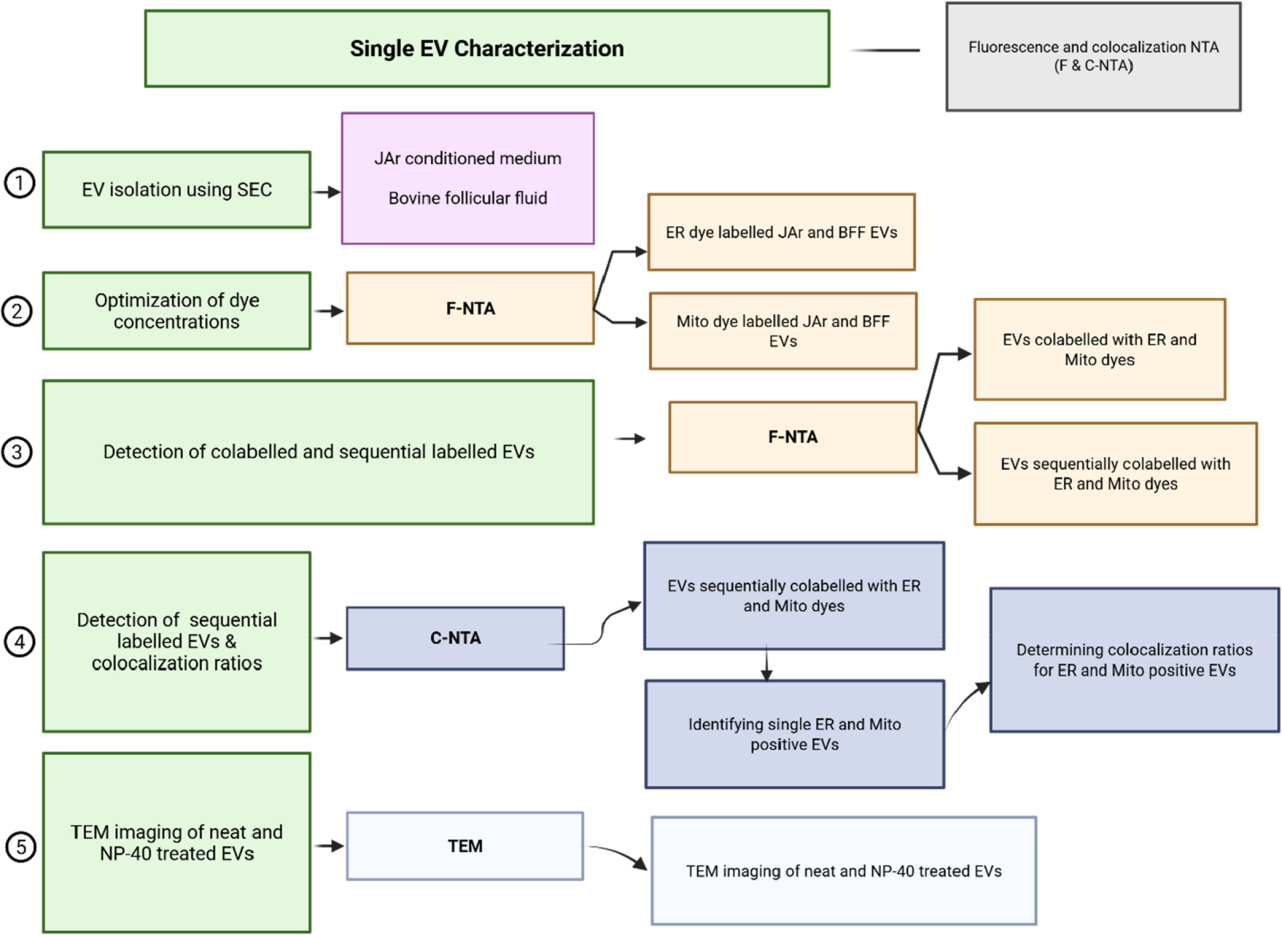
Experimental workflow for the detection of organelle-specific dyes labeled EVs using fluorescence and colocalization NTA (the figure was created with Biorender.com).

### 3.2 Sequential Labeling of JAr and BFF EVs Reveals Distinct ER- and Mito-Positive EV Population

To investigate whether detection of fluorescent nanoparticles is affected by sequences of EV labeling, EVs were colabeled and/or sequentially labeled with ER and Mito organelle-specific dyes and detected using QUATT ZetaView NTA. First, JAr EVs were colabeled with 480 nM ER and 50× Mito dye concentrations, whereas BFF EVs were colabeled with 640 nM ER and 40× Mito dye before being detected using fluorescence-NTA (Figure S3A). As shown in the previous Results section, heterogeneity in EV size distributions was also observed for JAr and BFF EVs coincubated with the respective concentrations of ER and Mito dye (Figure S3B,C). Interestingly, the coincubation approach affected the detection of Mito-specific colabeled vesicles for both EV types. In the case of BFF EVs, coincubation of ER and Mito dyes resulted in a very strong fluorescence signal that exceeded the saturation point (Figure S3E). This might be linked to the spillover effect of Mito dye. In contrast, a weak/low fluorescence signal was observed for ER-positive JAr EVs colabeled with Mito dye (Figure S3D). Since there was little/no Mito fluorescence signal when both EVs were colabeled and incubated together, the EV labeling sequence was changed. As a result, we modified the concentration of the ER dye used in the labeling of BFF EVs. The section below describes the detection of Mito- and ER-positive vesicles sequentially labeled with Mito and ER organelle-specific dyes using QUATT ZetaView NTA (Figure S4A).

The presence of EVs with heterogeneity in size distribution was observed for individual/single (Figure S4B,C) and sequential Mito- and ER-labeled (Figure 2A,D) JAr and BFF EVs measured in scatter (total) and fluorescence modes of NTA. The mean size shift toward larger particles was detected for Mito and ER fluorescent nanoparticles (f-NPs) of JAr and BFF EVs. Further statistical analysis on the mean particle sizes of fluorescent and total nanoparticles (t-NPs) of JAr and BFF EVs labeled individually (Figure S4D,E) and sequentially (Figure 2B,E) showed a significant shift toward larger particles (*p* < 0.05). Of interest here is the increase in the mean particle size for fluorescent Mito- and ER-positive EVs during sequential labeling than a single labeling approach. The labeling efficiency for individually ER-positive JAr EVs was 22.63 ± 19.49%, compared to that of Mito-positive EVs, 15.95 ± 5.54% (Figure S4F). Sequentially labeling approach of JAr EVs with Mito and ER dyes provided Mito- (14.09 ± 2.35%) and ER-positive fluorescent particles (16.75 ± 6.63%), highlighting that the sequential labeling approach did not affect the detection of ER- and Mito-positive vesicles (Figure 2C), whereas BFF EVs labeled only with ER dye (Figure S4G) was 54.81 ± 17.83%, compared to 60.17 ± 22.57% when sequentially colabeled with Mito organelle dye (Figure 2F). Regarding Mito dye labeling efficiency, BFF EVs (20.75 ± 9.84%) provided higher Mito-positive fluorescent particles (Figure S4G) than EVs sequentially colabeled with ER organelle dye (13.27 ± 2.47%) (Figure 2F). Overall, sequential labeling of JAr and BFF EVs revealed a distinct ER- and Mito-positive EV population.

**Figure 2:**
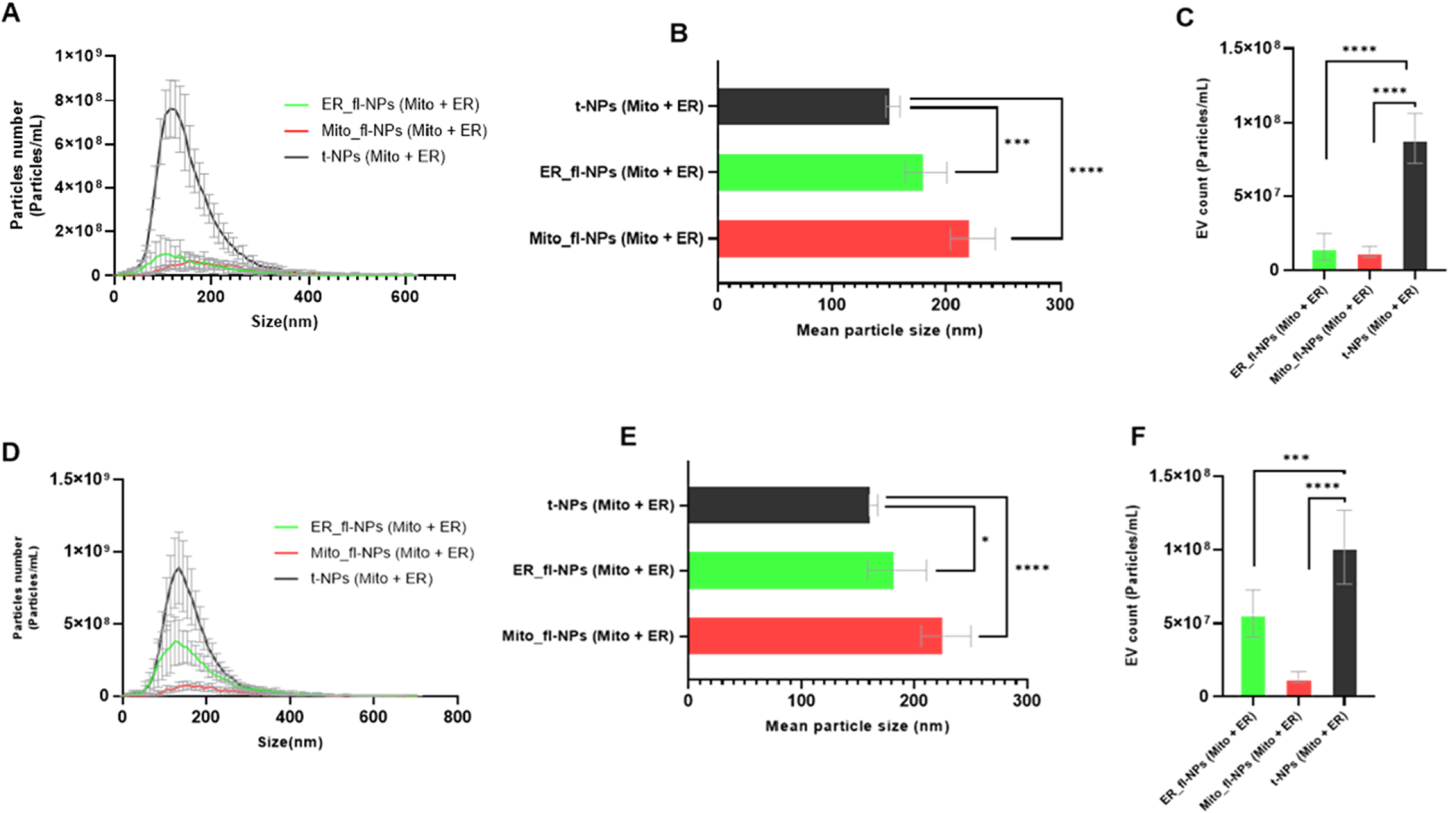
Sequential labeling of JAr and BFF EVs with Mito and ER organelle-specific dyes and detection with ZetaView Quatt NTA. (A and D) Size distributions of sequentially colabeled Mitochondria labeled (Mito_fl-NPs) and Endoplasmic Reticulum (ER _fl-NPs) with respective total particles originated from JAr cells and BFF. (B and E) Particle mean size and (C, F) concentration for respective ER and Mito fl and t-NPs of JAr and BFF EVs. The mean particle size and concentration for fl*-*NPs of JAr and BFF EVs sequentially colabeled with Mito and ER organelle-specific dyes were significantly different (*p* < 0.05) from the corresponding t-NPs of JAr and BFF EVs. Thus, they are marked with an asterisk (*) symbol. NP only as a control, and fl and t-NPs of EVs diluted in 1× PBS and measured in the scatter and fluorescence modes of NTA (mean ± SD, *n* = 9).

### 3.3 Colocalization-NTA (C-NTA) Identifies the Subcellular Origin and Single- and Double-Positive ER and Mitochondria-Derived Vesicles

#### 3.3.1 Colocalization-NTA (C-NTA) Can Detect the Origin of ER and Mito Organelle-Derived Vesicles

To determine the colocalized vesicles derived from the ER and mitochondrion compartments of the cells, JAr and BFF EVs were colabeled sequentially with Mito and ER-specific organelle dyes. EVs were sequentially colabeled with Mito and ER organelle-specific dyes and detected using Colocalization-NTA (C-NTA). As previously described, JAr EVs were sequentially labeled with 50x Mito and 640 nM ER dye concentrations, whereas BFF EVs were labeled with 40x Mito and 384 nM ER dye concentrations. Due to the differences in the software and optical configurations between the NTA Instruments, the concentrations of ER dye were modified accordingly. C-NTA was used in standard scattering and fluorescence modes to compare size profiles, mean particle size, and concentrations between neat and labeled EVs. EV heterogeneity in particle size distribution was also observed for JAr and BFF EVs sequentially labeled (Figure 3A­(i),B­(i)) with Mito and ER organelle-specific dyes, as well as single ER/Mito labeling (Figure S5B,C). In a subsequent study, we also labeled JAr and BFF EVs with CMDR, a lipophilic dye that is nonfluorescent until bound to membranes, to verify the presence of lipid-bilayer vesicles and to check the quality of EV preparations (Figure 3A­(ii),B­(ii)).

**Figure 3:**
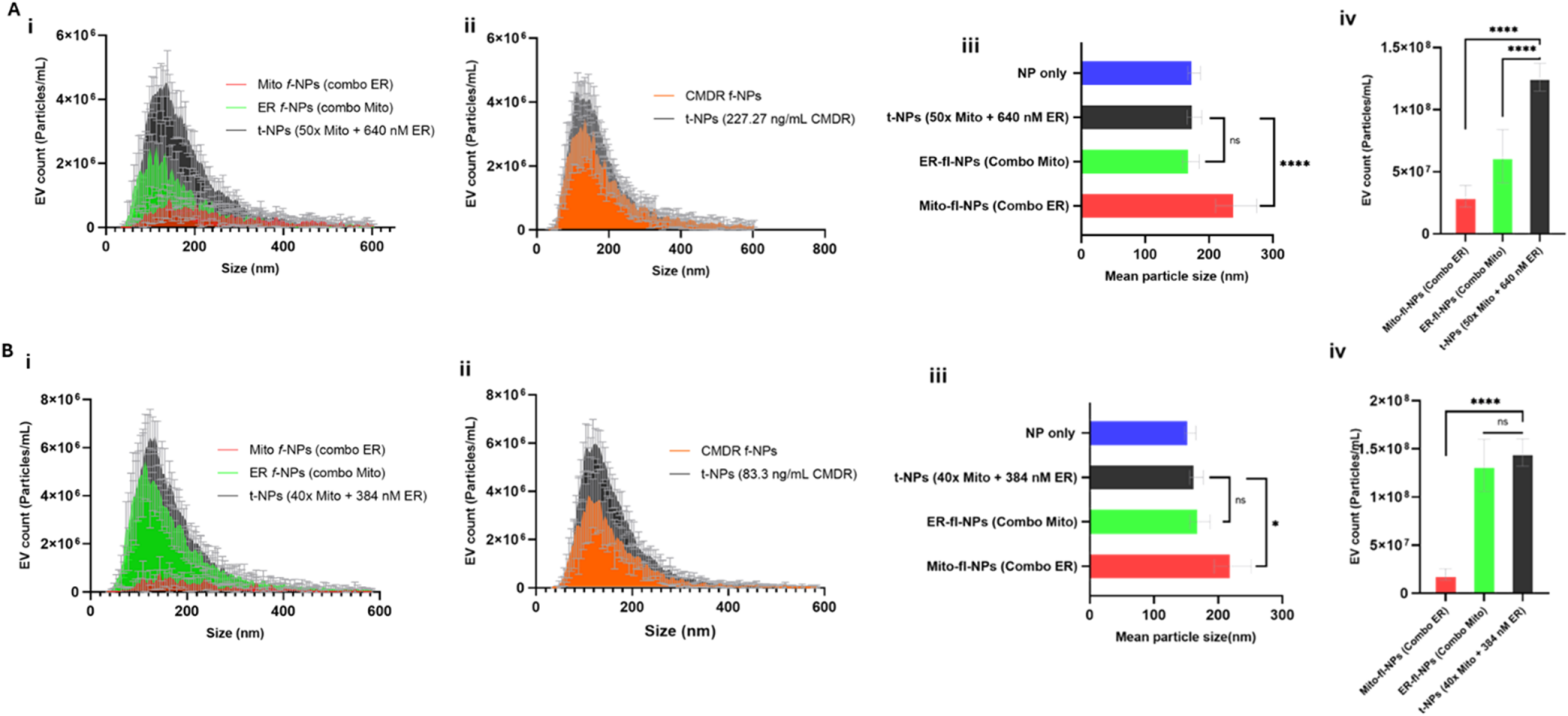
Detection of Mito- and ER-labeled JAr and BFF EVs by ZetaView C-NTA. Particle size distribution of JAr and BFF EVs labeled with (A­(i), B­(i)) a mix of Mito and ER, as well as (A­(ii), B­(ii)) CMDR with respective total particles. Particle mean size (A­(iii), B­(iii)) and concentration (A­(iv), B­(iv)) for the respective fluorescent ER and Mito and total NPs of JAr and BFF EVs detected at the sequential colabeling method. The mean particle size for Mito fl*-*NPs of JAr and BFF EVs sequentially colabeled with Mito and ER organelle-specific dyes was significantly different (*p* < 0.05) from the corresponding t-NPs of JAr and BFF EVs, except for the ER fl-NPs of JAr and BFF EVs. The concentrations for Mito and ER fl*-*NPs of JAr and BFF EVs were also significantly different from t-NPs of JAr and BFF EVs, except for the ER fl-NPs of BFF EVs. Thus, they are marked with an asterisk (*) symbol. The fl and t-NPs of EVs were diluted in 1× PBS and measured in scatter and fluorescence modes of C-NTA (mean ± SD, *n* = 9).

Quantitative determination on the mean particle size of ER fluorescent and total NPs of JAr EVs showed a significant shift in the means toward smaller particle size with only ER-labeled EVs (153.53 ± 10.46 nm)­(*p* < 0.05), except for those EVs colabeled sequentially with Mito and ER (171.36 ± 12.51 nm) dyes (*p* > 0.05) (Figures S5D­(i) and 3A­(iii)). Interestingly, no significant difference in the mean particle size of ER fluorescent and total NPs of BFF EVs occurred with only ER-labeled EVs (166.07 ± 12.08 nm) and sequentially colabeled with Mito and ER (171.74 ± 15.01 nm) dyes (*p* > 0.05) (Figures S5E­(i) and 3B­(iii)). Regarding Mito-labeled JAr EVs, a significant difference in the mean particle size for fluorescent Mito vesicle shifting toward a larger particle size was observed for JAr EVs labeled only with Mito (247.81 ± 23.14 nm) and sequentially coincubated with ER (242.45 ± 30.58 nm) organelle dyes (*p* < 0.05) (Figures S5D­(i) and 3A­(iii)). In case of BFF EVs, a significant difference in the mean particle size for fluorescent Mito vesicle shifting toward larger particle size was observed only for BFF EVs colabeled sequentially with Mito and ER dyes (222.61 ± 27.34 nm) (*p* < 0.05) than Mito dye only labeled EVs (204.30 ± 78.06 nm) (Figures S5E­(i) and 3B­(iii)). The polydispersity index (PDI), which is a measure of sample heterogeneity based on size, was also calculated for Mito- and ER-positive JAr and BFF vesicles (Scheme S1). Table 1 shows the PDI for fluorescent and total NPs of the JAr and BFF EVs.

**Table 1:** PDI of Fluorescent and Total NPs of JAr and BFF EVs

**total NPs**	**PDI (JAr)**	**PDI (BFF)**	**fluorescent NPs**	**PDI (JAr)**	**PDI (BFF)**
NP only	1.22	1.19			
t-NPs (ER)	1.22	1.19	f-NPs (ER)	1.34	1.27
t-NPs (Mito)	1.23	1.19	f-NPs (Mito)	1.24	1.23
t-NPs (Combo_Mito)	1.23	1.19	f-NPs (Combo_Mito)	1.34	1.27
t-NPs (Combo_ER)	1.23	1.19	f-NPs (Combo_ER)	1.26	1.22
t-NPs (CMDR)	1.23	1.19	f-NPs (CMDR)	1.32	1.23

JAr and BFF EVs were labeled with the CMDR membrane as well as ER and Mito organelle-specific dyes before being detected using C-NTA. JAr EVs labeled only with ER and in combination with Mito dye showed a difference in the concentration of ER-fluorescent NPs. After ER dye labeling, the concentration of ER-positive fluorescent NPs of JAr EVs was 67.11 ± 25.49 and 49.25 ± 14.97% colabeled with Mito dye (Figures S5D­(ii) and 3A­(iv)). In contrast, there were no significant differences observed in the concentration of ER-fluorescent NPs between BFF EVs labeled only with ER (96.27 ± 13.72%) and in combination with Mito (90.13 ± 9.28%) dye (Figures S5E­(ii) and 3B­(iv)) (*p* > 0.05). Regarding Mito EV labeling, both JAr (25.74 ± 4.46%) and BFF EVs (14.21 ± 7.45%) with Mito dye alone and in combination with ER dyes (23.90 ± 5.58 and 13.31 ± 3.21%) showed comparable labeling efficiency (Figures 5SD­(ii),E­(ii) and 3A­(iv),B­(iv)). Compared to the 84.81 ± 16.28 and 63.62 ± 16.80% purity of JAr and BFF EVs, it is clear that not all EVs were fluorescently labeled with CMDR membrane dye (Figure 3A­(ii),B­(ii)). Neither the EV only nor the free dyes (i.e., blank negative controls) emitted fluorescence when excited at the respective wavelengths (λ_500_ and λ_660_ nm), indicating that the free dye alone did not generate a false-positive fluorescence signal.

#### 3.3.2 Colocalization-NTA Analysis Revealed Single and Double-Positive ER and Mito Organelle-Derived Vesicles

In a subsequent study, the colocalization ratios for vesicles positive for ER and Mito were also determined. Using C-NTA, the same volume of fluorescently labeled particles was illuminated rapidly with one laser after the other with two different wavelengths. Following this, the fluorescence signals were detected in series with the corresponding filters (Figure 4A and Table S2). Note that the rapid switching and link radius (i.e., nearest-neighbor algorithm) were used to ensure insignificant distortion due to diffusion and almost identical illumination of the measuring volume. C-NTA analysis of the dual-stained JAr and BFF EVs showed 15.3 ± 3.62 and 8.0 ± 5.73% of the particles with ER, are double-labeled and have Mito-positive particles, respectively (Figure 4B­(ii),C­(ii)). Similarly, 37.67 ± 7.60 and 72.22 ± 6.71% of Mito-positive particles were also double-labeled and have ER-derived particles from JAr and BFF, respectively (Figure 4B­(ii),C­(ii)). It is worth noting that there were also differences in the ratios of single ER- and Mito-positive JAr and BFF EVs. Among the dual-labeled JAr EVs, 68.42 ± 7.04 and 18.87 ± 6.03% were single ER- and Mito-positive particles, respectively, whereas only 12.67 ± 2.52% were colocalized in the mixture of Mito and ER-labeled vesicles (Figure 4B­(i)). Differences in the ratios of single ER and Mito positive were also observed for BFF EVs. In the dual-labeled BFF EVs, 90.21 ± 6.27 and 2.32 ± 1.56% were single ER- and Mito-positive particles, respectively, while 7.46 ± 4.76% were ER/Mito double-positive or colocalized particles in the mix of Mito- and ER-labeled vesicles (Figure 4C­(i)). Overall, these results showed that the C-NTA used in this study can detect fluorescently labeled EVs and analyze the colocalization of dual fluorescence signals.

**Figure 4:**
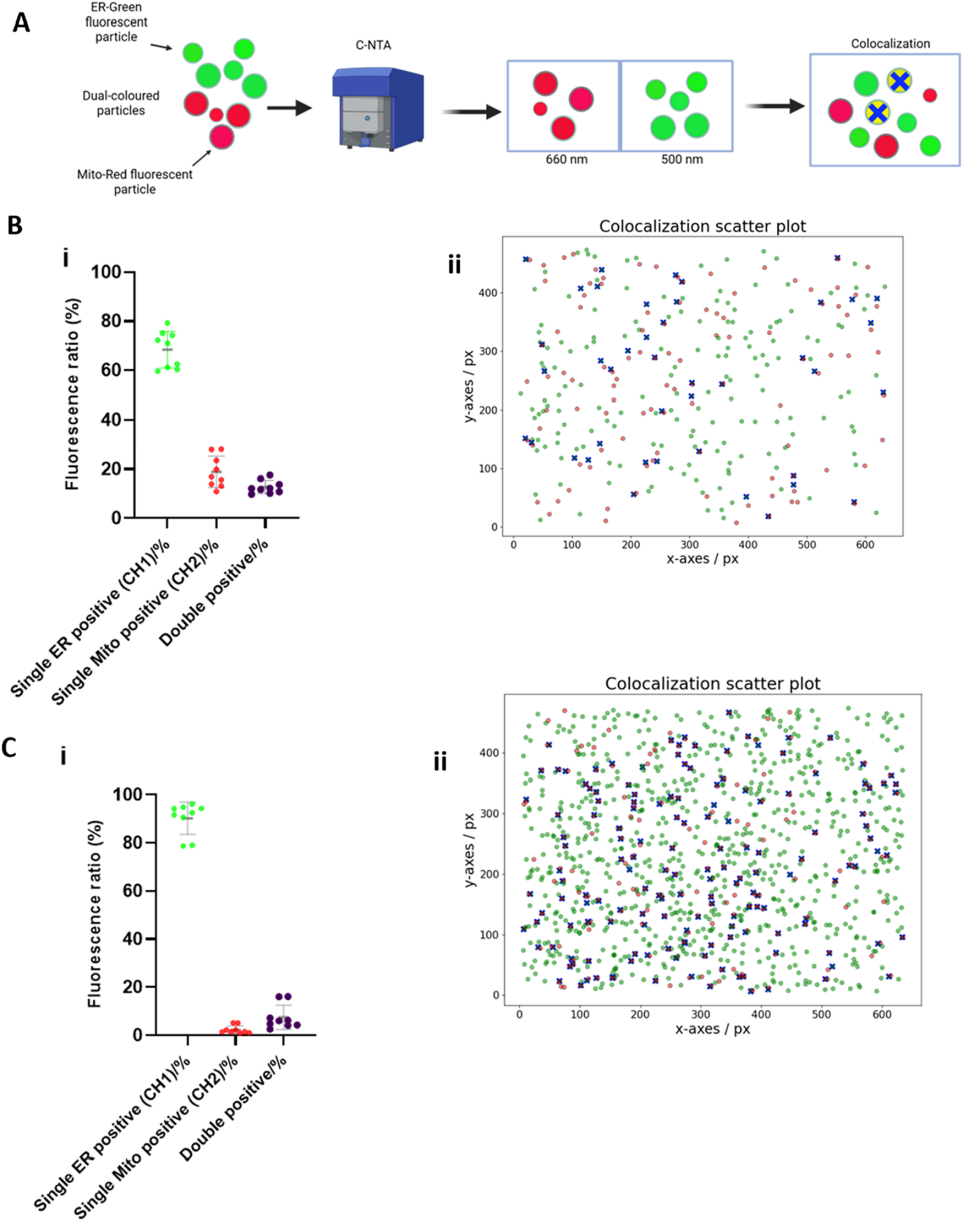
Detection of ER-Mito contacts on individual JAr and BFF EVs using C-NTA. (A) Schematic of EVs sequentially coincubated with Mito and ER-organelle-specific dyes and detection. (B­(ii) and C­(ii)) Representation of the *x*–*y* centroid coordinates of the scattering events of JAr and BFF EVs double-stained with ER and Mito organelle-specific dyes. Colocalization of JAr and BFF EVs was shown using 2 fluorescence channels (CH1-Green and CH2-Red) and an overlay of fluorescent images. Each green dot represents an EV positive for ER, while each red dot indicates a Mito-positive particle. Double-positive EVs are highlighted by a purple cross. (B­(i) and C­(i)) Data represent the single ER- and Mito-positive particles as well as colocalization (%) of double-positive JAr and BFF EVs (mean ± SD, *n* = 9) (Figure 4A was created with Biorender.com).

### 3.4 Detergent Treatments Affect the Membrane Integrity of ER- and Mito-labeled EVs

To confirm the fluorescence signals derived from membranous particles, EVs were treated with a nonionic 0.5% NP-40 detergent to disrupt their membranes. NP-40 detergent treatment of neat (unlabeled) JAr and BFF EVs led to a significant decrease in particle concentration (Figure 5A­(i)). However, TEM analysis of NP-40-treated JAr (Figure 5A­(iii)) and BFF (Figure 5B­(iii)) EVs indicated that the membrane structure appeared to be damaged as opposed to that of untreated EVs (Figure 5A­(ii),B­(ii)).

**Figure 5:**
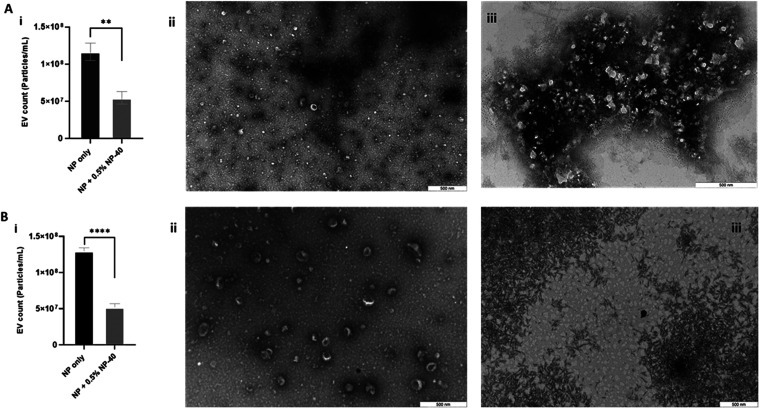
Concentration and TEM visualization of JAr and BFF EVs before and after NP-40 detergent treatment (A­(i) and B­(i)). Concentration for JAr and BFF EVs treated without (A­(ii) and B­(ii)) and with 0.5% NP-40. The EV membrane was disrupted (A­(iii) and B­(iii)), and TEM images showed the particle aggregates after detergent treatment. Scale bars are 500 nm for the panels (mean ± SD, *n* = 9).

As shown in Figure 6A,E and C,G, NP-40 treatment of ER-derived JAr and BFF EVs resulted in differential reactions. NP-40 detergent treatment of ER-labeled BFF EVs led to a significant decrease in fluorescent particle concentration both in single (96.2 to 52.3%) and colabeled sequentially with Mito dyes (90.1 to 22.8%) (Figure 6C,G). On the contrary, a small fraction of ER-positive JAr EVs were affected by NP-40 treatment (Figure 6A,E). In contrast to JAr EVs, mitochondrial-derived BFF EVs may have a lipid composition that renders them more susceptible to detergent treatment, leading to membrane disruption (Figure 6B,E and D,G). The detergent treatment of CMDR-positive fluorescent NPs of JAr and BFF EVs also resulted in complete membrane disruption (Figure 6F,H). For both EV types, no colocalization events were observed in the detergent controls (i.e., after the addition of 0.5% NP-40).

**Figure 6:**
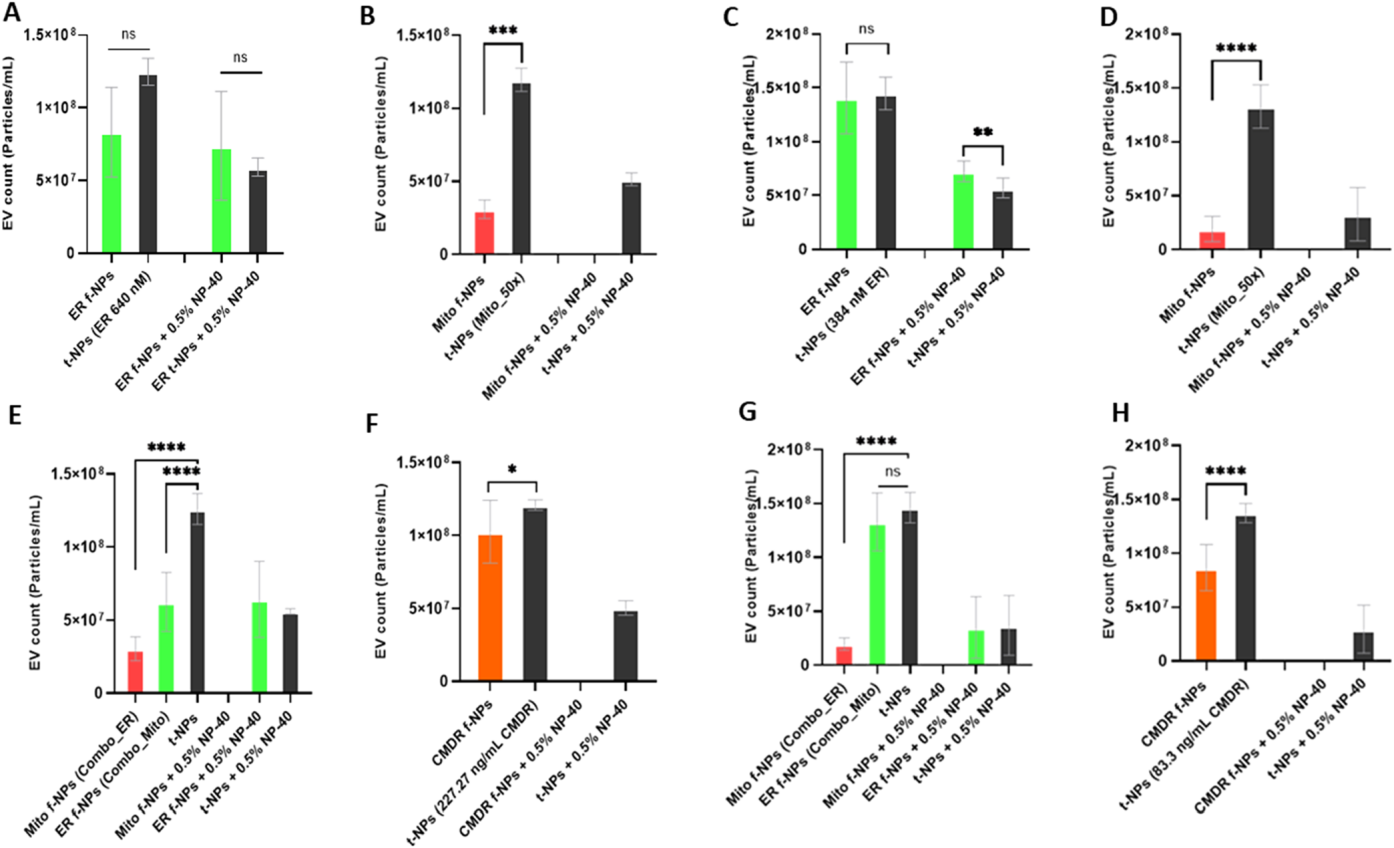
Concentration of fluorescent and total particles derived from JAr cells and BFF following the NP-40 detergent treatment. JAr and BFF EVs with (A, C) ER, (B, D) Mito, and a (E, G) mix of both dyes, as well as (F, H) CMDR, were treated with 0.5% NP-40 and measured in terms of their respective particle concentration. A significant drop in the particle concentration was observed for EVs labeled with organelle-specific and membrane dyes after NP-40 treatment (mean ± SD, *n* = 9).

## 4 Discussion

We first demonstrated the detection ability of F-NTA using JAr and BFF EVs labeled with different concentrations of ER and Mito dyes. Successive increase in ER-specific organelle dye concentration led to smaller particle mean sizes and higher labeling efficiency for JAr and BFF EVs. A similar trend was also observed for Mito dye-labeled JAr and BFF EVs. Differences in the sizes and subtypes of EVs could also lead to a differential dye affinity. In this case, EVs stained with ER dye are the brightest with the highest labeling efficiency. This means that each EV can be stained with a large number of ER dye molecules. Detection of fluorescent particles was unmeasurable at the lowest concentration of Mito dye-labeled EVs. At the highest concentrations of both ER and Mito dye, the measurement for fluorescently labeled particle concentration was saturated. It is important to note that excessive dye concentrations reduce efficiency due to the formation of dye aggregates, or nanoparticles that can be mistaken for labeled EVs. Subsequently, the above scenarios impacted distinguishing the fluorescence signal (detection) of bright particles from the noise and background. In line with previous observations, at the highest dye concentrations, detection of fluorescently labeled particles was challenging as invalid trajectories would be generated/recorded during measurement.^23,34,35^ Thus, regardless of the analytical technology used, such as NTA, flow cytometry, or other fluorescence-based techniques, the concentration and conditions of the dye application should always be optimized. This includes verifying the optimal dye concentration, the incubation conditions, and, in cases where multiple dyes are used, the sequence of labeling. Establishing such optimization steps should be part of the standard experimental practice for reliable and reproducible results.

C-NTA analysis revealed that sequential labeling of JAr and BFF EVs resulted in the detection of ER and Mito-derived/positive vesicles. With respect to the 85% purity of EVs isolated from JAr cells, it was identified that ER and Mito dyes could only label 50–57% and 25% EVs, respectively. However, it is interesting to note that almost 90–96% of BFF EVs were labeled with ER dye, implicating differences in glycosignatures in EVs secreted from JAr cells.^14,36,37^ The observed increase in ER-positive vesicles in BFF EVs could be explained in part by post-translational modifications (PTMs). Due to cells undergoing glycosylation, the outer membrane of exosomes is enriched in densely arranged glycans.^37^ For instance, O-linked glycosylation is one of the PTMs where glycans are added to serine or threonine in the secretory compartments (ER and Golgi). Hence, this explains why ER dyes/probes have a high affinity for the ER membranes in different cell types.^38^ This finding broadly supports other studies conducted in different contexts, providing evidence that the exosomes (small EVs) of differentiated cardiomyocyte cell lines carry an ER resident protein (ENPL) as their cargo.^39^ On the contrary to ER dye labeling efficiency, both JAr and BFF EVs were labeled lower with Mito dye, while the former had slightly higher Mito-positive vesicles (25%) than BFF EVs (7.5 to 14%). There are some similarities between Mito-positive fluorescent particles originating from EV samples used in this study and those described by Rosina et al., who also reported comparable labeling efficiency of MitoTracker Green dye-labeled brown adipose tissue and adipocytes derived EVs using flow cytometry.^40^ Furthermore, a study by D’souza et al. demonstrated that microvesicles (not exosomes) derived from human brain endothelial cell line transfer polarized mitochondria to recipient brain endothelial cells in culture exposed to oxygen-glucose deprivation (i.e., in vitro model of cerebral ischemia).^41^ The presence of distinct ER and Mito populations in EV populations may also have biological significance related to the ER-Mito contact sites (MERCS). MERCS play crucial roles in facilitating calcium signaling, lipid metabolism, organelle crosstalk, regulating mitochondrial dynamics, and quality control.^42^ Recent studies have shown that MERCS regulates Mito-derived vesicles under stress conditions, thereby influencing EV formation by promoting the incorporation of distinct organelle-derived components into subpopulations.^43,44^ For instance, in the context of cellular stress, MERCS could mediate the selective budding of EVs enriched in ER markers for protein quality control, while mitochondrial components are released separately to reduce oxidative damage. In addition, stress-induced mitochondrial EV release can serve as a mechanism for maintaining cellular homeostasis, where damaged mitochondria are packaged into EVs to prevent apoptosis or propagate signals.^45,46^ The low Mito signals observed in our study may reflect such selective release under experimental conditions, while the high ER signals observed in BFF EVs could be a result of ER stress responses in follicular environments. This connection underscores the biological relevance, as distinct EV roles could mediate intercellular communication in reproductive or pathological contexts such as choriocarcinoma progression. One interesting finding is that Mito-positive EVs appear to have a particle mean larger than that of ER-positive ones. This finding was also reported by Rai et al.^47^ Various studies have demonstrated that cells secrete large EVs containing mitochondria or mitochondrial components (free mitochondrial DNA, functional mitochondria, proteins, and cardiolipin) under cellular stress or impaired mitochondrial quality control.^18,48,49^ The inclusion of mitochondrial components, which range from 0.5 to 1 μm in diameter, could contribute to the larger size of Mito-positive vesicles. Overall, the presence of distinct ER and Mito-derived vesicles in JAr and BFF suggests that intracellular organelles may contribute to EV biogenesis and secretion.

In the present study, the colocalization ratios for vesicles positive for ER and Mito were also determined using C-NTA. C-NTA analysis revealed that only 13% and 8% were colocalized in the mixture of Mito- and ER-labeled JAr and BFF vesicles, respectively. This finding suggests that vesicles may communicate with one another at the ER–Mitochondria contact sites during EV biogenesis and before being secreted to the extracellular space. Although a study showed the colocalization of CD81, a common EV tetraspanin marker, with mitochondria,^18^ no previous study investigated the colocalization of fluorescently labeled organelle-derived vesicles or their contribution to EV heterogeneity. This study suggests that EVs may be involved in the trafficking landscape of intracellular vesicular organelles, which could contribute to EV biogenesis and secretion. Studies have shown that the communication at the contact sites between ER and Mito is crucial for maintaining various homeostatic processes.^50,51^ Additionally, the low colocalization ratios highlight the functional diversity of EV populations associated with distinct biogenesis pathways. This is crucial to their roles in intercellular communications. In biological systems, EVs derived from JAr and BFF may carry cargoes with specialized functions, such as mito EVs that are involved in energy transfer^52,53^ or stress responses.^54,55^ Furthermore, EVs may carry ER markers for protein folding^56,57^ or lipid metabolism.^58,59^ A low colocalized ratio implies specialized EVs that integrate multiple organelle functions, possibly used for targeted delivery in reproductive or pathological contexts. To better understand these subpopulations, advanced techniques such as nanoflow cytometry are required.^60,61^ This will aid in biomarker discovery for diseases and inform EV-based therapeutics where specific markers are coexpressed. Overall, these results showed that the C-NTA used in this study can detect fluorescently labeled EVs and analyze the colocalization of dual fluorescence signals, as well as hinting at the numerous aspects of ER–mitochondria contacts in the broader context of EV biology.

Furthermore, the membrane integrity of JAr and BFF EVs was confirmed using the NP-40 detergent. Nonionic NP-40 was used to confirm the disruption of the EV lipid bilayer membrane, both in the absence and presence of ER, Mito, and CMDR dyes within samples. This relates directly to assessing membrane integrity, as NP-40 solubilizes lipid membranes, disrupting vesicle structure if intact.^23,62^ Both CMDR and Mito-labeled EVs were more susceptible to detergent treatment, leading to complete membrane disruption. Interestingly, ER-labeled EVs showed resilience against detergent-induced disruption, retaining membrane integrity to a certain degree. The difference in detergent treatment resistance of the EV membrane is influenced by several factors. These factors include the nature of fatty acid chain saturation,^63^ lipid composition,^64^ lipid packing,^65^ dye’s selectivity and binding affinity.^66^ These factors collectively contribute to effective EV labeling and detergent resistance. Comparably, various studies reported the effect of detergents on the membrane integrity of EVs.^67,68^ Overall, NP-40 treatment of ER and mitochondrial-derived EVs resulted in differential reactions and also demonstrated that the particles under study were enclosed with membranous structures.

Despite the detection of ER- and Mito-positive particles in JAr and BFF EVs, this study was constrained by limitations. These limitations might include fluorophore stability issues (e.g., coincubation labeling), the inherent heterogeneity nature of the EV membrane, and the measurement technique applied. Regarding fluorophore stability, we confirmed that the selected fluorophores are well-suited for our study and the instrument used, demonstrating stable incorporation into EV membranes under single and dual label conditions without significant photobleaching during detection. However, for future multidye applications, researchers should be cautious about potential interactions such as collisional quenching (e.g., coincubation labeling), which occurs when dye–dye contact reduces fluorescence efficiency, which impacts the ability to quantify fluorescence and colocalization studies. In C-NTA, the fast switch between the fluorescence channels, as well as the software-defined link radius, ensures low distortion due to particle diffusion, whereas the use of the shift-pro tool ensures identical illumination of the measuring volume. However, fluorescent particles may be slightly off the focal plane, resulting in weak fluorescence signals and a low colocalization ratio for the studied EV samples. Additionally, integrating a highly sensitive camera into NTA might also improve the detection of faintly labeled EV samples.^69^ Although advanced sensitive cameras could mitigate faint labeling issues, the employed C-NTA successfully determined ER- and Mito-positive colocalized populations, with prospects for multifunctional upgrades in future C-NTA systems. Hence, when choosing effective EV membrane labeling and detection methods, it is important to take into account the aforementioned limitations. Future studies should include colocalization analysis using NTA and compare the results with those obtained by techniques such as flow cytometry or super-resolution microscopy. This would help us to further validate and expand the current findings. Advances in EV detection methods might offer an in-depth understanding of how organelle-derived vesicles mediate intercellular communication and their implications in health and disease.^70,71^ Further improving our understanding of the organelle-derived vesicles biogenesis as they can provide us with emerging therapeutic approaches, e.g., using mito-derived vesicles to rejuvenate and restore damaged mitochondria.^72^

In this study, the sequence of labeling was found to cause differences in the detection of ER- and Mito-positive EV populations. When both JAr and BFF EVs were simultaneously labeled with ER and Mito organelle-specific dyes, there was little/no Mito fluorescence signal observed for both EV types. On the other hand, sequential labeling of JAr and BFF EVs resulted in the detection of distinct ER- and Mito-positive EV populations. Various factors may explain the differences in the detection of Mito fluorescence signals following different staining sequences (coincubation vs sequential). It is possible that the ER dye can interfere with the Mito dye’s fluorescence due to direct chemical or physical interactions between the dyes, leading to collisional quenching.^73^ Specifically, collision quenching occurs when a quencher molecule (e.g., the ER dye) transiently collides with the excited Mito dye, transferring energy and preventing light emission. This process is diffusion-dependent and preferably occurs in high-concentration solutions or when molecules are in close proximity, resulting in increased collisions. Due to a number of reasons, collisional quenching is not relevant for sequential labeling: First, Mito and ER dyes typically exhibit distinct spectral properties, which minimize overlaps with energy transfer mechanisms, such as Forster resonance energy transfer (FRET). Second, dyes bind to specific or membrane-associated sites in EVs, reducing their mobility and collision frequency compared with free solutions (i.e., without EVs). The concentrations used in EV staining are typically low, which further reduces the collision rate. During coincubation, where EVs are exposed to both Mito- and ER organelle-specific dyes simultaneously, structural changes may occur as a result of these dyes integrating into EV membranes. The simultaneous insertion of both dyes’ hydrophobic tails increases membrane rigidity and may induce local changes in membrane curvature. This can inhibit or hinder the efficient binding or retention of the Mito dye, especially if the ER dye molecules occupy binding sites or alter the lipid packing density, reducing accessibility to mitochondrial-derived components within EVs. It is also possible that coincubation may promote EV aggregation more than sequential staining because the presence of multiple dye molecules increases the number of hydrophobic interactions between EVs. This aggregation can lead to a heterogeneous dye distribution, where Mito dye is less uniformly incorporated, resulting in lower overall fluorescence signals. Studies on PKH dye labeling, for example, have demonstrated that dye concentrations and simultaneous exposure can contribute to aggregation, altering EV morphology and influencing quantitative detection.^74,75^ Sequential staining, in which one dye is applied and stabilized before a second dye is applied, allows for better preservation of the integrity of the EV and controlled dye incorporation. Overall, the fluorescence signal differences can be attributed to labeling impacts on EV structure and environment during coincubation, which supports the use of sequential labeling for accurate analysis in F-NTA. Furthermore, the ER dye’s surface binding might block/limit Mito dye access to mitochondria by altering the local environment within the EVs, including pH, ionic strength, and membrane potential. These ER dye structural changes and modified environments can affect Mito dye’s fluorescence during the coincubation process. Given that organelle-specific dyes can be susceptible to changes in pH or membrane potential, future studies should examine or discuss these vulnerabilities. Recent studies on EV labeling also demonstrated the effect of surface interaction on the detection of fluorescent EVs.^76^ Moreover, the impact of dye labeling on EV bioactivity warrants future investigation.^77^ The use of lipophilic dyes, for example, could alter EV membrane fluidity, potentially impairing fusion or cargo release in applications involving EV tracking in live-cell uptake assays or in vivo imaging.^76,78,79^ These studies indicate that careful consideration should be given to dye selection, proper single stain controls, and optimal staining conditions when labeling EVs with multiple dyes. Comparison of NTA fluorescence analysis with other fluorescence-based EV detection methods, such as flow cytometry or super-resolution imaging, would be useful. It will allow for a clearer assessment of the relative sensitivity, specificity, practicality, and limitations of these methods. Such a study could be planned as a continuation of the current work in future investigations.

## 5 Conclusions

The current study aimed to identify the subcellular origin of vesicles colabeled with organelle-specific fluorescent dyes and detect them using F-NTA and C-NTA. We used EVs secreted from bovine follicular fluid (BFF) and human choriocarcinoma JAr cells as model systems. Successive increase in ER and Mito-specific organelle dye concentrations led to smaller particle mean sizes and higher labeling efficiency for JAr and BFF EVs. This observed size shift underscores the need to emphasize optimal dye concentrations to maintain EV size after labeling. The influence of different staining sequences on the detection of Mito fluorescence signals was observed for both EV types. This study also determined the colocalization ratios for vesicles double-positive for ER and Mito, as well as single ER/Mito-positive EVs, using C-NTA. TEM analysis revealed the cup-shaped morphology of EVs, and the membrane integrity of fluorescently labeled EVs was confirmed using detergent treatment. Our data indicated that C-NTA can detect fluorescently labeled EVs and analyze the colocalization of dual fluorescence signals, contributing to the EV research on ER–mitochondria communications in the context of EV biology. Considering the observed colocalization ratios of the studied EV groups, super-resolution microscopy could be used to investigate the ER–mitochondria contact dynamics in live cells to elucidate interactions associated with EV biogenesis. Moreover, lipidomic profiling may also provide insight into the differential NP-40 resistance detected in ER- and Mito-labeled EVs. Further research is needed to explore the mechanism behind the secretion of multiple types of EVs from different subcellular origins.

## Supplementary Material



## Data Availability

Raw data for NTA are available in the Supporting Information. The analyzed data are either presented in the article or can be provided upon request.

## Notes

Tables for fluorescence and colocalization measurement parameters for ZetaView PMX-420 Quatt, figures for the Effect of ER and Mito dye concentration on the physical characteristics of fluorescent and total NPs of JAr and BFF EVs, figure for coincubation labeling of JAr and BFF EVs with Mito and ER organelle-specific dyes and detection with ZetaView Quatt NTA, figure for labeling of JAr and BFF EVs with Mito and ER organelle-specific dyes and detection with ZetaView Quatt NTA, figure for Detection of Mito- and ER-labeled JAr and BFF EVs by ZetaView C-NTA and calculation of the PDI for JAr EV (PDF)
