# Supplementary material for: Detection of Organelle-Specific Dyes Labeled Extracellular Vesicles with Colocalization-Fluorescence Nanoparticle Tracking Analysis
Source: ACS Omega. 2026 Feb 3;11(6):10530–44. doi: 10.1021/acsomega.5c12069 (PMC12917633; doi:10.1021/acsomega.5c12069)
Supplement: Supplementary file 1 [file ao5c12069_si_001.pdf]

Supporting Information

Detection of Organelle-Specific Dyes Labelled Extracellular Vesicles with Colocalization-Fluorescence Nanoparticle Tracking Analysis

Getnet Midekessa[1,2], Kasun Godakumara[2], Mohammad Mehedi Hasan[3], Aneta Andronowska[4], Alireza Fazeli*[1,2,5]

[1]Department of Pathophysiology, Institute of Biomedicine and Translational Medicine, University of Tartu, Tartu, Estonia; [2]Institute of Veterinary Medicine and Animal Sciences, Estonian University of Life Sciences, Tartu, Estonia; [3]Research Department of Maternal and Fetal Medicine, Elizabeth Garrett Anderson Institute for Women's Health, University College London, London, UK; [4]Institute of Animal Reproduction and Food Research, Polish Academy of Sciences, Olsztyn, Poland; [5]Academic Unit of Reproductive and Developmental Medicine, Department of Oncology and Metabolism, Medical School, University of Sheffield, Sheffield, UK. [*Corresponding author; Email: alireza.fazeli@emu.ee]

Table S1. Measurement parameters for ZetaView PMX-420 Quatt

Measurement parameters	Scatter mode λ = 488 nm	Fluorescence mode λ = 488F500 nm	Fluorescence mode λ = 640F660 nm
Positions	11	11	11
Number of cycles	3	3	3
Number of frames	30	30	30
Sensitivity	72	90	90
Shutter	100	100	100
Min Brightness	30	30	30
Max Area	1000	1000	1000
Min Area	10	10	10
Trace length	15	15	15
Frame rate	30	30	30
Tracking radius			

Table S2. Additional measurement parameters used for colocalization measurement on a PMX-420 Quatt

Measurement parameters	Scatter mode λ = 488 nm	Fluorescence mode λ = 488F500 nm	Fluorescence mode λ = 640F660 nm
Positions	11	5	5
Number of cycles	1	1	1
Video length	30	30	30
Sensitivity	77	90	90
Shutter	200	100	100/200 colo
Min Size/nm	1	1	1
Max Size/nm	1000	1000	1000
Frame rate	30	30	30
Trace length	12	12/3 colo	12/3 colo
Link Radius		15	15

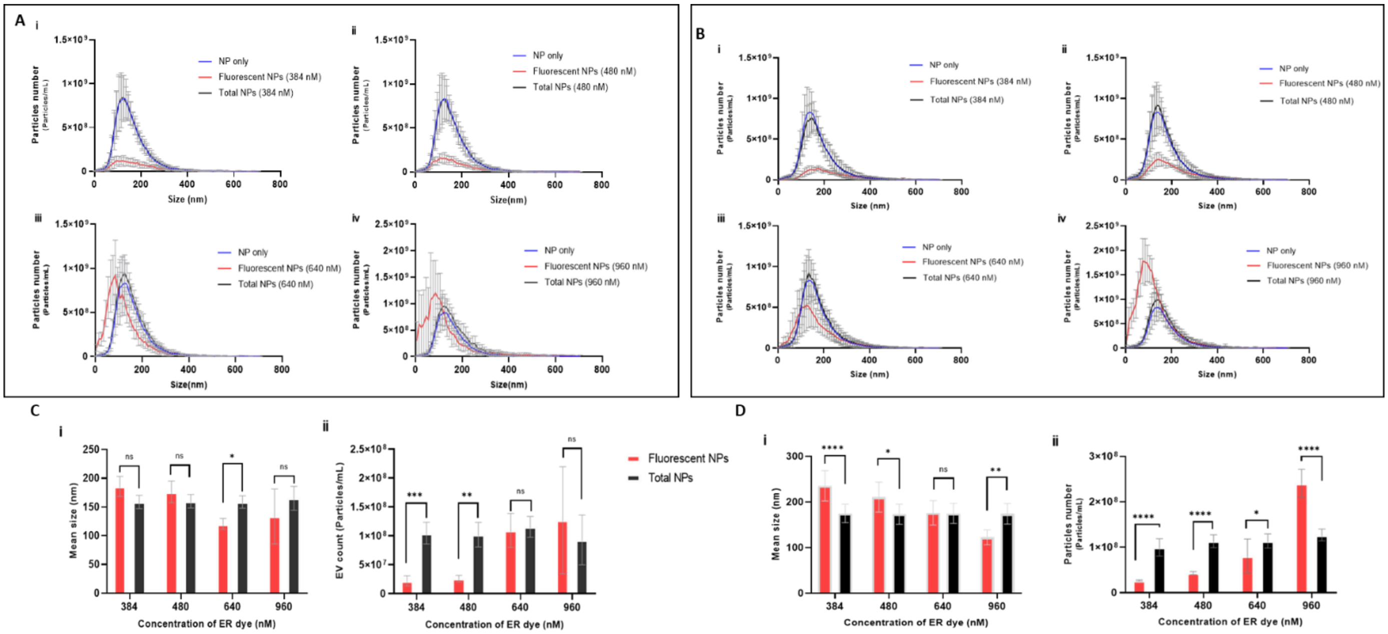

Figure S1. Effect of ER dye concentration on the physical characteristics of fluorescent and total NPs of JAr and BFF EVs. (A) Size profile distribution for fluorescent NPs derived from JAr cells and (B) BFF measured at different concentrations of 384, 480, 640 and 960 nM respectively. Particle means size and concentration for fluorescent and total NPs originating from JAr cells (C: i-ii) and BFF (D: i-ii) measured in the scatter and fluorescent modes. NP only as a control, fluorescent and total NPs of EVs diluted in 1 XPBS and measured in scatter and fluorescent modes of ZetaView Quatt NTA (mean ± SD, *n* = 9).

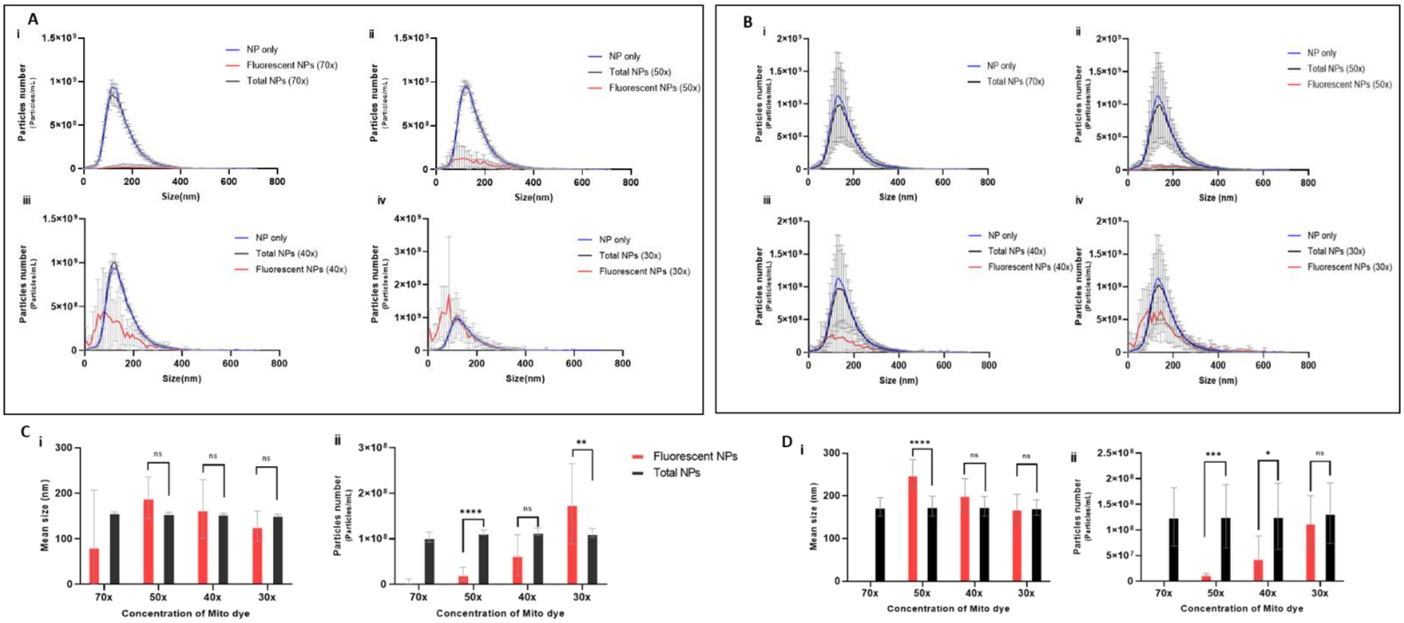

Figure S2. Effect of Mito dye concentration on the physical characteristics of fluorescent and total NPs of JAr and BFF EVs. (A) Size profile distribution for fluorescent NPs derived from JAr cells and (B) BFF measured at different concentrations of 70x, 50x, 40x and 30x respectively. Particle means size and concentration for fluorescent and total NPs originating from JAr cells (C: i-ii) and BFF (D: i-ii) measured in

the scatter and fluorescent modes. NP only as a control, fluorescent and total NPs of EVs diluted in 1 XPBS and measured in scatter and fluorescent modes of ZetaView Quatt NTA (mean ± SD, *n* = 9).

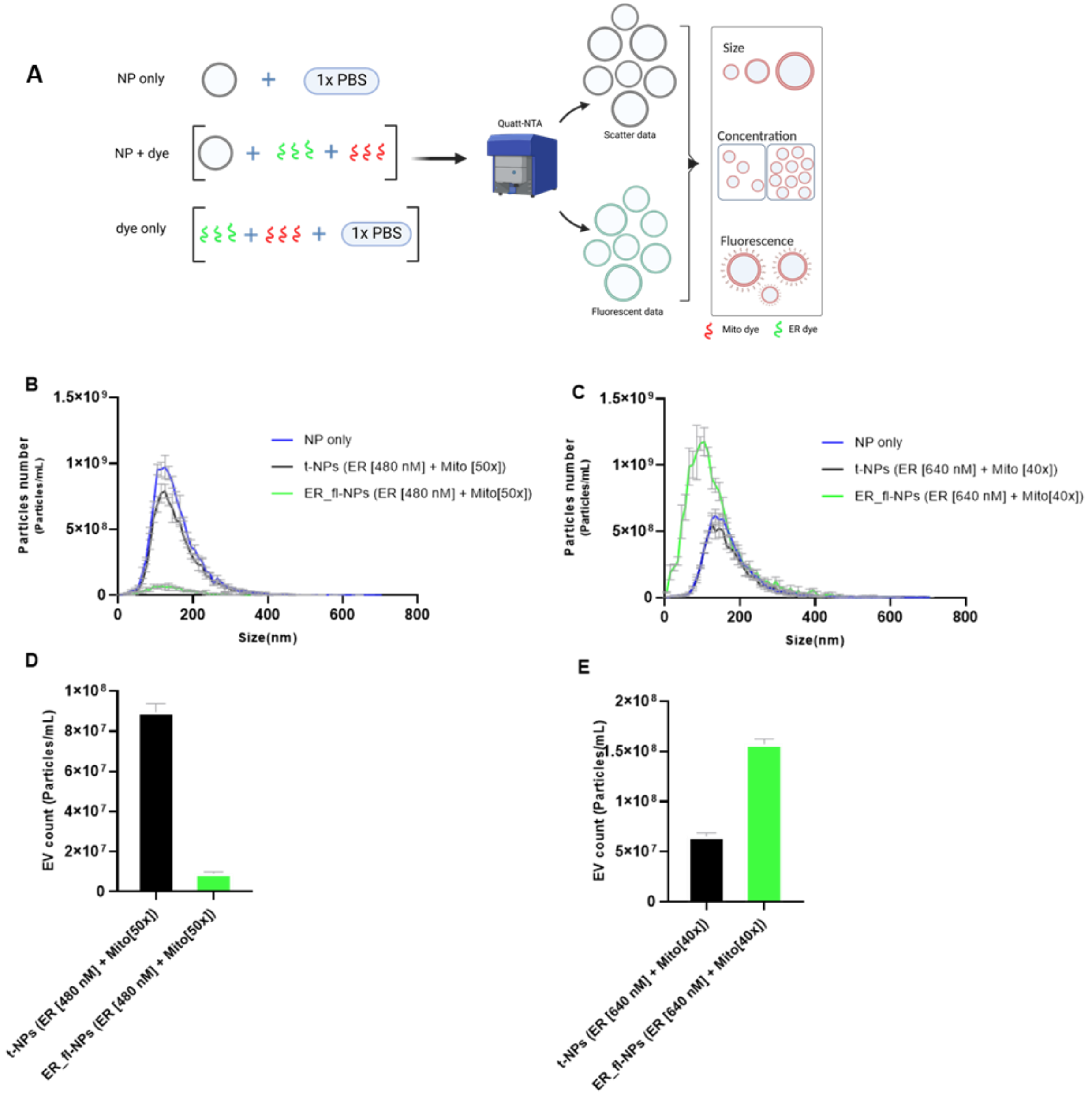

Figure S3. Coincubation labeling of JAr and BFF EVs with Mito and ER organelle-specific dyes and detection with ZetaView Quatt NTA . (A) Schematic of EVs coincubation with ER and Mito-organelle specific dyes and detection. (B and C) Size distributions of unlabeled JAr and BFF EVs (NP only) and colabeled at the same time with ER and Mito dyes. (D and E) concentration for respective ER fl and t-NPs of JAr and BFF EVs. The fluorescence signal was only observed for ER though both samples were incubated with the respective Mito dye. NP only as a control, fl and t-NPs of EVs diluted in 1xPBS and measured in the scatter

and fluorescent modes of NTA . Data presented as mean ± SD. (The figure S3.A was created with Biorender.com).

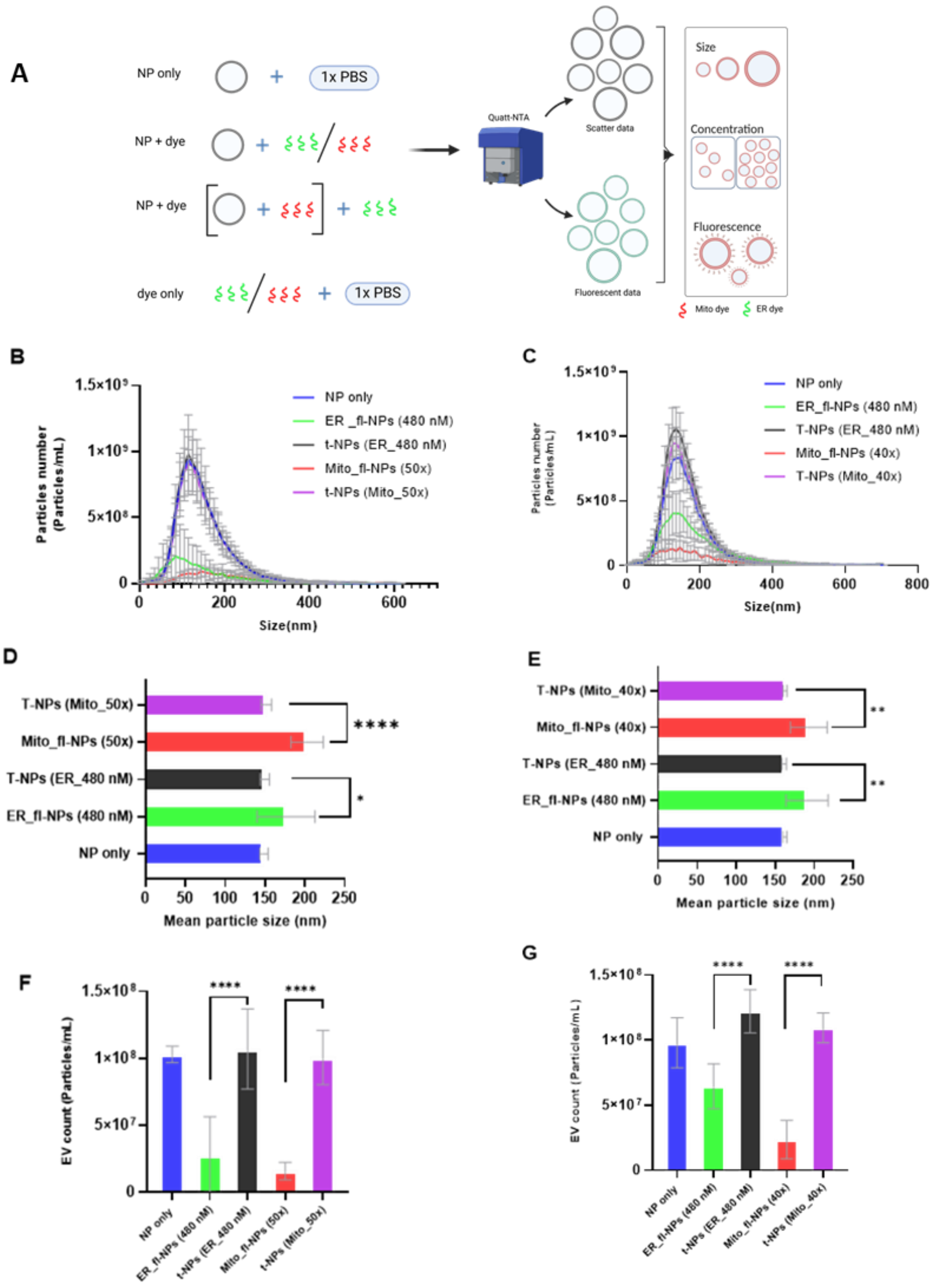

Figure S4. Labeling of JAr and BFF EVs with Mito and ER organelle-specific dyes and detection with ZetaView Quatt NTA. (A) Schematic of EVs individually and/or sequentially coincubated with Mito and ER-organelle specific dyes and detection. (B and C) Size distributions of unlabeled EVs (NP only) and individually Endoplasmic reticulum (ER _fl-NPs) or Mitochondria labelled (Mito_fl-NPs) with respective total particles originated from JAr cells and BFF. (D and E) particle means size and (F and G) concentration for respective ER and Mito fl and T-NPs of JAr and BFF EVs. NP only as a control, fl and T-NPs of EVs diluted in 1xPBS and measured in the scatter and fluorescent modes of NTA (mean ± SD, n = 9). (The figure S4.A was created with Biorender.com).

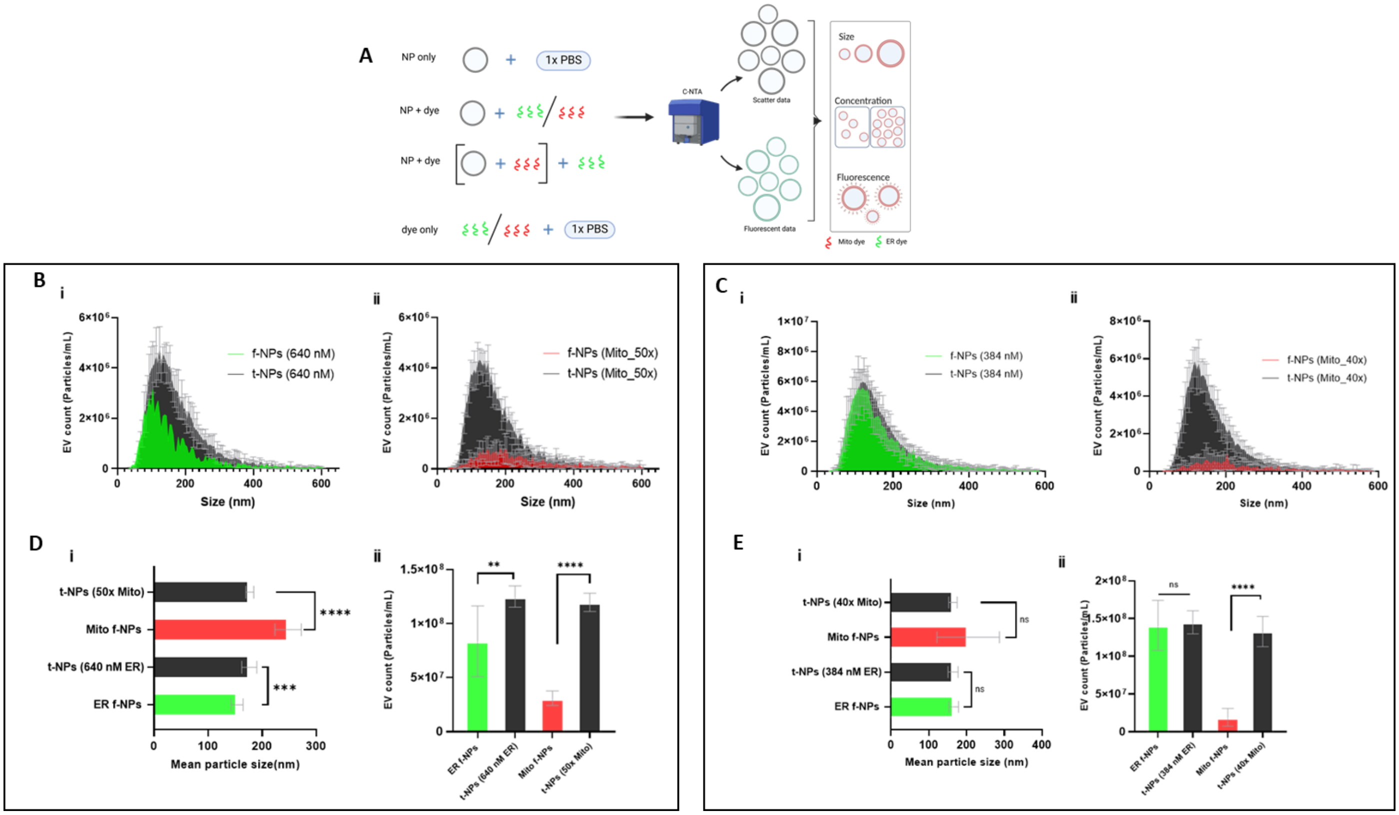

Figure S5. Detection of Mito and ER labeled JAr and BFF EVs by ZetaView C-NTA. (A) Schematic of EVs individually and/or sequentially coincubated with Mito and ER-organelle specific dyes and detection. Particle size distribution of JAr and BFF EVs labelled individually/separately with (B: i-ii, C: i-ii) Mito and ER with respective total particles. Particle means size (D-i, E-i) and concentration (D-ii, B-ii) for the respective fluorescent ER and Mito and total NPs of JAr and BFF EVs detected at individual labelling method. The mean particle sizes for fl-NPs of JAr EV individually labelled with ER and Mito organelle-specific dyes were significantly different ($p < 0.05$) than the corresponding t-NPs of JAr EVs, except those Mito and ER fl-NPs of BFF EVs. The concentration for Mito and ER fl-NPs of JAr and BFF EVs was also significantly different than t-NPs of JAr and BFF EVs, except those ER fl-NPs of BFF EVs. Thus, were marked with an asterisk (*)

symbol. The fl and t-NPs of EVs diluted in 1X PBS and measured in scatter and fluorescent modes of C-NTA (mean ± SD, *n* = 9). (The figure S5.A was created with Biorender.com).

Scheme S1. Determination of the PDI from the size distribution of the JAr EVs (i.e. NP only) shown in Figure 3

Ave concentration (**N**$_i$)	Size/nm (**M**$_i$)	(**M**^2_i)	(**N**$_i$**M**$_i$)	(**N**$_i$**M**^2_i)
0	33.5	1122.25	0	0
0	38.5	1482.25	0	0
30665	43.5	1892.25	1333927.5	58025846.25
22321	48.5	2352.25	1082568.5	52504572.25
137576.2222	53.5	2862.25	7360327.889	393777542.1
241646.5556	58.5	3422.25	14136323.5	826974924.8
516500.7778	63.5	4032.25	32797799.39	2082660261
733503.6667	68.5	4692.25	50245001.17	3441782580
1177204.444	73.5	5402.25	86524526.67	6359552710
1602130	78.5	6162.25	125767205	9872725593
2200968	83.5	6972.25	183780828	15345699138
2562019.333	88.5	7832.25	226738711	20066375924
2959109.333	93.5	8742.25	276676722.7	25869273569
3481377.444	98.5	9702.25	342915678.3	33777194310
3591664	103.5	10712.25	371737224	38474802684
3849211.889	108.5	11772.25	417639489.9	45313884659
3647788.222	113.5	12882.25	414023963.2	46991719826
4157600.778	118.5	14042.25	492675692.2	58382069522
4130041.889	123.5	15252.25	510060173.3	62992431400
3907310.222	128.5	16512.25	502089363.6	64518483217
3772920.556	133.5	17822.25	503684894.2	67241933371
3787069.556	138.5	19182.25	524509133.4	72644514982
3957463.667	143.5	20592.25	567896036.2	81493081190
3671405.444	148.5	22052.25	545203708.5	80962750712
3757489.222	153.5	23562.25	576774595.6	88534900426
3245397.444	158.5	25122.25	514395494.9	81531685949
3415936.111	163.5	26732.25	558505554.2	91315658106
3094956.667	168.5	28392.25	521500198.3	87872783419
3190741.444	173.5	30102.25	553593640.6	96048496646
2762640.333	178.5	31862.25	493131299.5	88023936961
2742758.333	183.5	33672.25	503296154.2	92354844290

2457484.444	188.5	35532.25	463235817.8	87319951651
2326635.111	193.5	37442.25	450203894	87114453489
2390519.444	198.5	39402.25	474518109.7	94191844780
2035024.111	203.5	41412.25	414127406.6	84274927245
1995706.556	208.5	43472.25	416104816.8	86757854310
1875344.444	213.5	45582.25	400386038.9	85482419303
1670083.556	218.5	47742.25	364913256.9	79733546630
1597664.111	223.5	49952.25	357077928.8	79806917094
1466082	228.5	52212.25	334999737	76547439905
1263452	233.5	54522.25	295016042	68886245807
1306895.333	238.5	56882.25	311694537	74339147075
1338980.222	243.5	59292.25	326041684.1	79391150081
1113875.556	248.5	61752.25	276798075.6	68784321776
1054504.444	253.5	64262.25	267316876.7	67764828235
868296.6667	258.5	66822.25	224454688.3	58021536934
831990.1111	263.5	69432.25	219229394.3	57766945392
889009.2222	268.5	72092.25	238698976.2	64090675101
874063.5556	273.5	74802.25	239056382.4	65381920599
820116	278.5	77562.25	228402306	63610042221
714804.8889	283.5	80372.25	202647186	57450477231
504863.6667	288.5	83232.25	145653167.8	42020938920
580163.5556	293.5	86142.25	170278003.6	49976594044
495652.4444	298.5	89102.25	147952254.7	44163748018
615178.7778	303.5	92112.25	186706759.1	56665501373
388557.5556	308.5	95172.25	119870005.9	36979896817
451688.3333	313.5	98282.25	141604292.5	44392945699
539818.2222	318.5	101442.3	171932103.8	54760375053
435494.7778	323.5	104652.3	140882560.6	45575508358
370130.7778	328.5	107912.3	121587960.5	39941645024
427850.5556	333.5	111222.3	142688160.3	47586501453
435818.6667	338.5	114582.3	147524618.7	49937083419
367525	343.5	117992.3	126244837.5	43365101681
370443.2222	348.5	121452.3	129099462.9	44991162836
277594.4444	353.5	124962.3	98129636.11	34688826365
285788.6667	358.5	128522.3	102455237	36730202465
277656.5556	363.5	132132.3	100928157.9	36687385413
256752.2222	368.5	135792.3	94613193.89	34864961948
173863.6667	373.5	139502.3	64938079.5	24254372693
256119.2222	378.5	143262.3	96941125.61	36692216044
190495.4444	383.5	147072.3	73055002.94	28016593629
173631.3333	388.5	150932.3	67455773	26206567811
242312.5556	393.5	154842.3	95349990.61	37520221305
286899.2222	398.5	158802.3	114329340.1	45560242012

149270.3333	403.5	162812.3	60230579.5	24303038828
262003.2222	408.5	166872.3	107028316.3	43721067199
119567.1111	413.5	170982.3	49441000.44	20443853684
114375.8889	418.5	175142.3	47866309.5	20032050526
127731.5556	423.5	179352.3	54094313.78	22908941885
104827.4444	428.5	183612.3	44918559.94	19247602936
118968.5556	433.5	187922.3	51572868.83	22356838639
145508.8889	438.5	192282.3	63805647.78	27978776551
98810.22222	443.5	196692.3	43822333.56	19435204932
61641.11111	448.5	201152.3	27646038.33	12399248193
127868.5556	453.5	205662.3	57988389.94	26297734840
143773.8889	458.5	210222.3	65920328.06	30224470413
105630.3333	463.5	214832.3	48959659.5	22692802178
74987.44444	468.5	219492.3	35131617.72	16459162903
91686.66667	473.5	224202.3	43413636.67	20556356962
67492.88889	478.5	228962.3	32295347.33	15453323699
91107.88889	483.5	233772.3	44050664.28	21298496178
98959	488.5	238632.3	48341471.5	23614808828
98892.66667	493.5	243542.3	48803531	24084542549
61010.77778	498.5	248502.3	30413872.72	15161315552
45389.66667	503.5	253512.3	22853697.17	11506836523
37232.11111	508.5	258572.3	18932528.5	9627190742
68290.33333	513.5	263682.3	35067086.17	18006948747
22573.33333	518.5	268842.3	11704273.33	6068665723
53423.88889	523.5	274052.3	27967405.83	14640936954
37857	528.5	279312.3	20007424.5	10573923848
46211.77778	533.5	284622.3	24653983.44	13152900168
37860.11111	538.5	289982.3	20387669.83	10978760205
37601.22222	543.5	295392.3	20436264.28	11107109635
45553.44444	548.5	300852.3	24986064.28	13704856256
29641.33333	553.5	306362.3	16406478	9080985573
37378.77778	558.5	311922.3	20876047.39	11659272467
29951	563.5	317532.3	16877388.5	9510408420
59791.55556	568.5	323192.3	33991499.33	19324167371
23138.33333	573.5	328902.3	13269834.17	7610249895
14421.22222	578.5	334662.3	8342677.056	4826238677
53721.44444	583.5	340472.3	31346462.83	18290661063
7429.111111	588.5	346332.3	4372031.889	2572940767
22965.11111	593.5	352242.3	13629793.44	8089282409
15221.66667	598.5	358202.3	9110167.5	5452435249
14718.66667	603.5	364212.3	8882715.333	5360718704

$\sum N_i = 116646733.8$

$\sum N_i M_i = 20635065094$

$\sum N_i M_i^{\ 2} = 4.46095E+12$

$\Rightarrow M_w = \sum N_i M_i^{\ 2} / \sum N_i M_i = 4.46095E+12/20635065094 = 216.1832235$

$\Rightarrow M_n = \sum N_i M_i / \sum N_i = 20635065094/116646733.8 = 176.9022109$

$\Rightarrow$ **PDI** $= M_w/M_n = 216.1832235/176.9022109 = 1.222049303 \approx 1.22$
